# Selective dorsal rhizotomy in ambulant children with cerebral palsy: an observational cohort study

**DOI:** 10.1016/S2352-4642(19)30119-1

**Published:** 2019-07

**Authors:** Jennifer Summers, Bola Coker, Saskia Eddy, Maria Elstad, Catey Bunce, Elli Bourmpaki, Mark Pennington, Kristian Aquilina, Stephanie Cawker, Richard Edwards, John Goodden, Sally Hawes, Kate McCune, Benedetta Pettorini, Jennifer Smith, Christine Sneade, Michael Vloeberghs, Hannah Patrick, Helen Powell, Christopher Verity, Janet L Peacock

**Affiliations:** a School of Population Health and Environmental Sciences, King's College London, London, UK; b Institute of Psychiatry, Psychology & Neuroscience, King's College London, London, UK; c Neurosurgery Department, Great Ormond Street Hospital for Children, London, UK; d Physiotherapy Department, Great Ormond Street Hospital for Children, London, UK; e Paediatric Neurosurgery, Bristol Royal Hospital for Children, Bristol, UK; f Paediatric Physiotherapy, Bristol Royal Hospital for Children, Bristol, UK; g Neurosurgery Department, Leeds General Infirmary, Leeds, UK; h Neuro-Rehabilitation and Spascticity Service, Leeds General Infirmary, Leeds, UK; i Paediatric Physiotherapy Department, Nottingham University Hospitals, Nottingham, UK; j Department of Neurosurgery, Nottingham University Hospitals, Nottingham, UK; k Department of Paediatric Neurosurgery, Alder Hey Children's Hospital, Liverpool, UK; l National Institute for Health and Care Excellence, Manchester, UK; m Children's and Adolescent Services, Addenbrooke's Hospital, Cambridge, UK

## Abstract

**Background:**

Selective dorsal rhizotomy (SDR) is an irreversible surgical procedure involving the division of selected sensory nerve roots, followed by intensive physiotherapy. The aim is to improve function and quality of life in children with cerebral palsy and a Gross Motor Function Classification System (GMFCS) level of II or III (walks with or without assistive devices, respectively). We assessed gross motor function before and after SDR and postoperative quality of life in a study commissioned by NHS England.

**Methods:**

We did a prospective observational study in five hospitals in England who were commissioned to perform SDR on children aged 3–9 years with spastic diplegic cerebral palsy. The primary outcome was score changes in the 66-item Gross Motor Function Measure (GMFM-66) and seven domains of the Cerebral Palsy Quality of Life Questionnaire ([CP-QoL] social wellbeing and acceptance, feelings about functioning, participation and physical health, emotional wellbeing and self-esteem, access to services, family health, and pain and impact of disability) from before to 24 months after SDR.

**Findings:**

From Sept 4, 2014, to March 21, 2016, 137 children underwent SDR. The mean age was 6·0 years (SD 1·8). The mean GMFM-66 score increased after SDR with an annual change of 3·2 units (95% CI 2·9 to 3·5, n=137). Of the seven CP-QoL domains, five showed significant improvements over time: feelings about functioning mean annual change 3·0 units (95% CI 2·0 to 4·0, n=133), participation and physical health 3·9 units (2·5 to 5·3, n=133), emotional wellbeing and self-esteem 1·3 units (0·2 to 2·3, n=133), family health 2·0 units (0·7 to 3·3, n=132), and pain and impact of disability −2·5 units (−3·9 to −1·2, n=133). 17 adverse events were reported in 15 children, of which none were severe and 15 (88%) resolved.

**Interpretation:**

SDR improved function and quality of life in the 24 months after surgery in children with cerebral palsy classified as GMFCS levels II and III. On the basis of these findings, an interim national policy decision was made that SDR would be funded for eligible children in England from 2018.

**Funding:**

National Institute for Health and Care Excellence, National Institute for Health Research Biomedical Research Centre, NHS England.

## Introduction

The worldwide incidence of cerebral palsy is 2–3 per 1000 livebirths,^1^ and approximately 80% of affected children have spasticity in their lower limbs.^2^ With no cure, treatment options aim to alleviate symptoms and improve quality of life. Medical treatments include intramuscular injections of botulinum toxin, oral baclofen, or both, to relieve muscle stiffness, anti-inflammatories for pain, and orthotic devices and physiotherapy to aid walking. Surgical interventions include operations on bony or soft tissue, orthopaedic surgery to restore lower-limb alignment, and neurosurgery such as selective dorsal rhizotomy (SDR).^3^ SDR is an irreversible neurosurgical procedure in which sensory nerve roots are divided. The aim is to reduce spasticity in the lower limbs and improve gross motor function and quality of life.^4^, ^5^, ^6^, ^7^ Intensive physiotherapy is provided for several months after SDR.^4^

Evidence from randomised controlled trials (RCTs) relating to SDR is limited. Three RCTs were done in Canada and the USA but were published between 1997 and 1998.^6^, ^8^, ^9^ These compared SDR plus physiotherapy with physiotherapy alone. Data from the 90 children assessed were summarised in a meta-analysis in 2002,^10^ which showed consistent overall improvement in function in children receiving SDR compared with those receiving physiotherapy alone (2·7 unit mean difference in scores in the 66-item Gross Motor Function Measure (GMFM-66) units at a median follow-up of 12 months; appendix). The RCTs were well reported and included robust methods of randomisation and allocation concealment, but follow-up was short (9–12 months) and no data were collected on quality of life. Two of the three RCTs reported no adverse events^8^, ^9^ and the third reported one spinal epidural abscess and one transient urinary retention.^6^

**Research in context**

**Evidence before this study**

Selective dorsal rhizotomy (SDR) for children with cerebral palsy has not been routinely available via the National Health Service (NHS) in England. Although some international evidence indicates positive long-term outcomes, NHS England judged that the evidence base was insufficient to support universal funding. We searched the Cochrane database, EMBASE, PubMed, Web of Science, and grey literature on Oct 15, 2018, for randomised controlled trials that compared SDR and non-SDR outcomes in patients with cerebral palsy. We found three small randomised controlled trials that involved 90 patients. A meta-analysis provided evidence of improved mean gross motor function 9–12 months after SDR with no suggestion of harm. All three studies, however, were done in the 1990s, since when surgical techniques have changed, and they captured no data on quality of life or long-term follow-up. A new randomised trial was impossible in the UK because of the lack of clinical equipoise among physicians and patients (including parents), yet firmer evidence of benefit without harm was needed to inform a policy decision. Therefore, in 2014, NHS England commissioned a robust evaluation of SDR with standardised outcomes and with 24 months of follow-up after surgery in five NHS paediatric neurosurgical centres in England.

**Added value of this study**

This assessment of SDR followed by intensive physiotherapy was done in a large contemporary group of 137 children. The study obtained prospective data on a wide range of validated outcomes collected in a standardised way, and particularly assessed gross motor function (66-item Gross Motor Function Measure) and quality of life (Cerebral Palsy Quality of Life Questionnaire for Children parent version), including pain and adverse events, over the 2-year follow-up. Assessment of multiple data points allowed patients' trajectories to be modelled statistically and showed clear improvement in function and quality of life, including reduced pain, with no evidence of harm. Furthermore, we compared our data with age-specific and severity-specific norm centiles for gross motor function that were developed in Canada for children with cerebral palsy who had not had SDR. These showed that the observed benefit of SDR among children in this study exceeded the expected function without SDR.

**Implications of all the available evidence**

This evaluation provides robust evidence that SDR plus intensive physiotherapy improves gross motor function, quality of life, and pain to greater degrees than would be expected without SDR. The evidence was used to inform a policy review in 2018 by NHS England, which resulted in funding of SDR being supported for eligible children aged 3–9 years with cerebral palsy.

SDR has not been routinely available via the National Health Service (NHS) in England and has been possible to obtain only through individual funding requests.^11^ Although the findings of several cohort studies (including the RCTs) have suggested positive long-term outcomes after SDR,^5^, ^6^, ^8^, ^9^, ^10^, ^12^, ^13^, ^14^ NHS England judged the evidence base to be inadequate to permit universal funding because studies were more than 20 years old and quality of life was not measured in the RCTs^4^ and requested further evidence for the effects of SDR on longer-term outcomes. A further RCT was not possible in the UK because of the lack of equipoise among physicians and parents who strongly believed that there was a need for SDR. Therefore, in 2014, NHS England commissioned a prospective evaluation of SDR in a series of eligible children with standardised outcomes and 2 years of follow-up. We report on the gross motor function and quality of life outcomes after SDR.

## Methods

### Study design and oversight

Five NHS paediatric neurosurgical centres in England (appendix) were commissioned by NHS England Commissioning through Evaluation programme to provide a package of care according to a standardised protocol. The Commissioning through Evaluation programme enables a limited number of patients to access treatments that are promising but not yet universally funded by the NHS to allow new data to be collected on clinical outcomes and patients' experiences. The SDR care package included surgery and physiotherapy for up to 24 months for eligible children. Data were collected in the Research Electronic Data Capture application^15^ and outcomes were reported to a national database.^4^ Planning and oversight of the study was provided by a multidisciplinary SDR steering committee, which included clinical and academic experts, representatives of patients, and NHS England commissioners. The study did not include a control group and, therefore, age-specific and severity-specific normalised GMFM-66 centiles developed in Canada and based on children who had not had SDR were used to provide comparative data.^16^, ^17^, ^18^

Ethics approval for the collation of data and its subsequent analysis was given by the UK Health Research Authority. Written informed consent for data collection and analysis was obtained from the parents or primary caregivers for all children.

### Selection of patients

Each centre had a multidisciplinary team that selected children for inclusion in the study according to a rigorous process. The teams were experienced in the delivery of SDR surgery and other accepted antispasticity treatments and the neurosurgeons performing surgery had previous experience and training in the SDR procedure. The assessment process was designed to balance functional abilities and impairments. A minimum list of assessments was provided by NHS England to be done for each patient before and after surgery (appendix).

Eligible children had spastic diplegic cerebral palsy that limited functional abilities and sufficient strength to rehabilitate after SDR surgery. Other inclusion criteria were age 3–9 years; MRI confirmation of no damage to key areas of the brain controlling posture or coordination (eg, lesions in the basal ganglia or cerebellum, which are associated with dyskinetic or ataxic cerebral palsy types) and typical cerebral palsy changes (white-matter damage of immaturity, namely periventricular leukomalacia); Gross Motor Function Classification System (GMFCS)^19^ level II (walks without assistive devices) or III (walks with assistive devices); no evidence of genetic or neurological progressive illness; mild to moderate lower-limb weakness with ability to maintain antigravity postures, no significant scoliosis or hip dislocation (Reimer's index^20^ <40%); confirmed financial or resource commitment for community-based postoperative physiotherapy; and no other relevant medical or personal contraindications. Patients with progressive neurological conditions and those with dystonia were excluded (appendix).

### Treatment and follow-up

SDR was performed with a single-level laminectomy or laminoplasty approach with neurophysiology-guided partial section of the dorsal (sensory) nerve roots. Intraoperative neurophysiology was mandated to guide the selection of the nerve rootlets being divided. Each nerve root from L1 to S1 was divided into smaller portions (rootlets) of approximately equal size, which were tested with neurophysiology to elicit a reflex motor response. The responses were recorded and graded for extent of spread to adjacent myotome levels.^21^ The rootlets responsible for the greatest neurophysiology responses were cut. The physiotherapy regimen after surgery was specified and recommended for 24 months after discharge from hospital. Follow-up assessments were done at 4–6 months, 12 months, and 24 months after surgery.

### Outcomes

The primary outcomes were change in gross motor function, measured with the GMFM-66 (a 66-item tool, with higher scores representing greater motor function, which is measured in five key motor domains: lying and rolling; sitting; crawling and kneeling; standing; and walking, running and jumping), which has been validated for assessment of children with cerebral palsy;^22^, ^23^ change in GMFM-66 centiles measured with the normalised version of GMFM-66, which is adjusted for age and GMFCS level; change in quality of life measured with seven domains of the parent or caregiver version of the Cerebral Palsy Quality of Life Questionnaire for Children ([CP-QoL] social wellbeing and acceptance, feelings about functioning, participation and physical health, emotional wellbeing and self-esteem, access to services, family health, and pain and impact of disability);^24^ and adverse events, measured for intensity, duration, outcomes, and relation to SDR. GMFCS was used to categorise the severity of involvement,^19^ and we used GMFM-66 as an objective assessment of neurological impairment during follow-up. Prespecified secondary outcomes were assessment of spasticity using the Modified Ashworth Scale,^25^ the Boyd and Graham Scale,^26^ gait analyses, and physiotherapy assessments (appendix).

### Statistical analysis

The study was originally intended to include 163 patients, but due to a delay in the launch of the Commissioning through Evaluation programme, the overall number treated was 137. GMFM-66 scores, GMFM-66 centiles, and CP-QoL scores were used to follow patients' trajectories based on within-patient changes from before to 24 months after SDR. We used a linear mixed model in which the patient was the random effect and time was modelled using time from SDR surgery to each assessment. Difference in changes over time was assessed by GMFCS level by fitting an interaction term in the model with a likelihood ratio test. All results were scaled to mean annual change with 95% CIs. Model fit and model assumptions were checked with residual plots. Changes in the Modified Ashworth Scale, Boyd and Graham Scale, and Gait Profile Score over time were analysed with non-parametric tests. Sensitivity analysis of all children and all children excluding the three children aged older than 9 years at the time of SDR surgery found no material difference in results. To account for the multiplicity of outcomes and sensitivity analysis, we adjusted for multiple testing using a Bonferroni correction for the eight-variable composite outcome of GMFM-66 score and the seven CP-QoL domain scores. The modified cutoff for significance for this composite outcome was 0·0064 (1 – 0·95^0·125^). Stata version 15 was used for all statistical analyses.

### Role of the funding source

The funders of the study had a role in the study design, data collection, data analysis, data interpretation, or writing of the report. The corresponding author had full access to the data and had final responsibility for the decision to submit for publication.

## Results

Between Sept 4, 2014, and March 21, 2016, 137 patients underwent SDR in the five study centres. No patients withdrew from the programme, but one patient did not complete the final assessment at 24 months. Most patients (61%) were boys and had GMFCS level III (62%; table 1). The mean length of hospital stay following SDR for all patients was 19·3 days (SD 7·1), although the time varied by centre (range 3–39 days). All patients had nerve rootlets cut during SDR in L2–S1, and 125 had rootlets cut in L1 (appendix). For most patients the percentage of rootlets cut for L1–S1 was between 60% and less than 70%.

**Table 1:** Baseline and operative characteristics

		**All patients (n=137)**
**Baseline characteristics**		
Age at SDR (years)		
	Mean (SD)	6·0 (1·8)
	Range	3–9*
Boys		83/137 (61%)
Girls		54/137 (39%)
GMFCS level		
	II	52/137 (38%)
	III	85/137 (62%)
Height before SDR (cm)		
	Mean (SD)	112·1 (12·7)
	Range	87·0–139·0
Weight before SDR (kg)		
	Mean (SD)	21·3 (7·0)
	Range	11·4–45·5
Body-mass index before SDR (kg/m^2^)		
	Mean (SD)	16·9 (4·0)
	Range	12·8–48·9
Body-mass index centile before SDR		
	Mean (SD)	55·2 (31·9)
	Range	0–100
Pregnancy duration (weeks)		
	Mean (SD)	32 (4·0)
	Range	26–42
Birthweight (kg)		
	Mean (SD)	1·9 (0·8)
	Range	0·8–4·2
Medication in previous 6 months		
	Oral baclofen	23/129 (18%)
	Diazepam	3/129 (2%)
	Botulinum toxin	15/129 (12%)
Previous surgery		
	Gastrocnemius or heel cord	0/129
	Bone	2/129 (2%)
	Adductor	0/129
	Hamstring	1/129 (1%)
SEN or education, health, and care plan		
	Learning difficulties	19/137 (14%)
	Behaviour, emotional, and social difficulty	20/137 (15%)
	Speech, language, and communication needs	6/137 (4%)
	Autistic spectrum disorder	5/137 (4%)
	Hearing impairment	1/137 (1%)
	Visual impairment	4/137 (3%)
	Physical disability other than cerebral palsy	17/137 (12%)
	No SEN disability	28/137 (20%)
	No SEN support	57/137 (42%)
**Operative characteristics**		
Length of stay in hospital after SDR (days)		
	Mean (SD)	19·3 (7·1)
	Range	3–39
Intraoperative neurophysiology		137/137 (100%)
Sphincter monitoring		114/122 (93%)
Mean proportion of nerve rootlet cut from L1 to S1 left and right†		64·6
Range of the mean proportion of the centre of nerve rootlet cut from L1 to S1 left and right†		57·1–66·0

SDR=selective dorsal rhizotomy. GMFCS=Gross Motor Function Classification System. SEN=special educational needs.

* Three children were aged 9 years at the time of the assessment before SDR and 10 years at time of surgery. A sensitivity analysis of all children and excluding the three children aged older than 9 years at the time for surgery showed no difference in results.

† Excludes rootlets with 0% cut.

GMFM-66 scores increased in almost all children from before surgery to 24 months after surgery (figure, appendix). Mean annual increases were significant (table 2), with the overall increase being 3·2 units per year (95% CI 2·9–3·5, p<0·0001). The estimated increase was higher in patients with GMFCS level II than in those with GMFCS level III (table 2), which was a significant difference (p_interaction_=0·006). Similarly, significant changes were seen for normalised GMFM-66 centiles over 2 years (table 2). The estimated norms for GMFM-66 calculated from the Canadian cohort who had not undergone SDR were lower than the modelled change and entirely below the 95% CIs estimated from the current SDR data. This was true for all children and in the GMFCS II and III subgroups (appendix).

**Figure:**
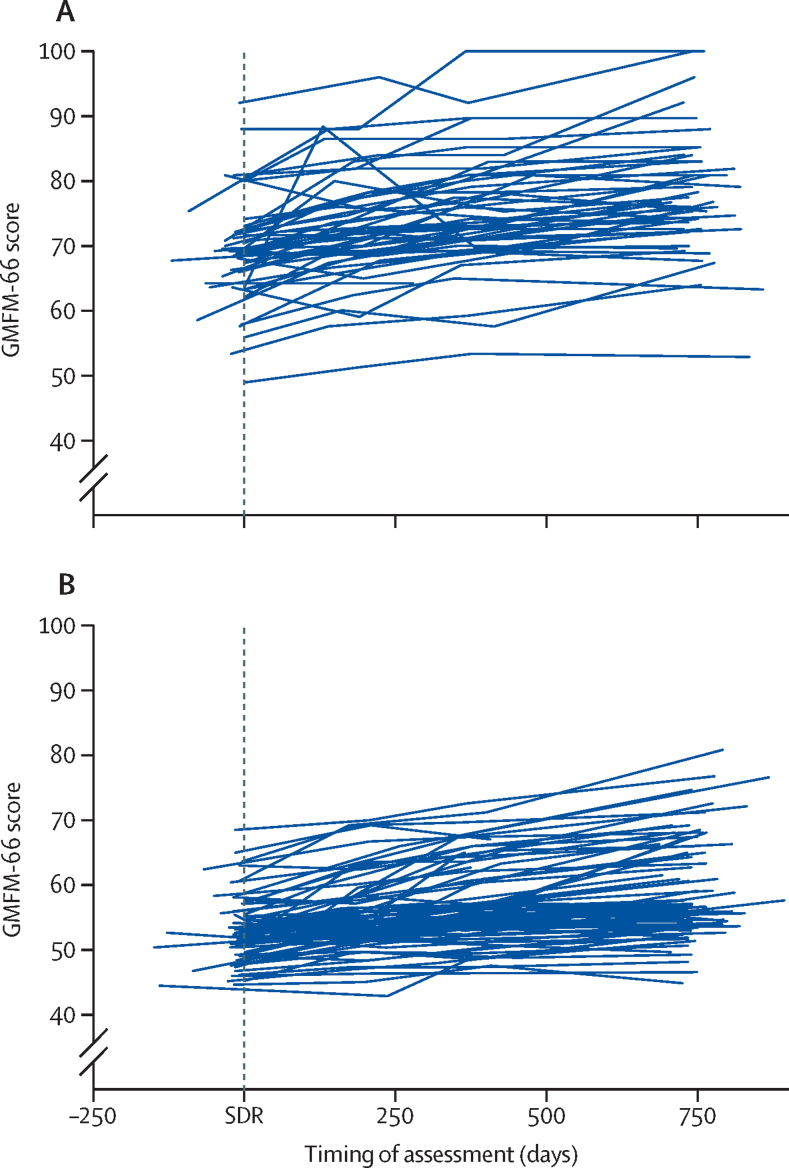
Observed trajectories of gross motor function for all patients by disease severity 137 children were assessed at baseline and all points before 24 months. (A) Gross Motor Function Classification System level II. (B) Gross Motor Function Classification System level III. GMFM-66=66-item Gross Motor Function Measure. SDR=selective dorsal rhizotomy.

**Table 2:** Primary outcomes

		**Before SDR**	**24 months after SDR**	**Modelled mean change per year (95% CI)**
GMFM-66*				
GMFCS level II		69·0 (7·9), n=52	77·6 (8·9), n=51	3·8 (3·2 to 4·2, p<0·0001)
GMFCS level III		52·8 (4·6), n=85	58·8 (7·5), n=82	2·9 (2·5 to 3·2, p<0·0001)
All patients		59·0 (9·9), n=137	66·0 (12·2), n=133	3·2 (2·9 to 3·5, p<0·0001)
**Normalised GMFM-66 centiles**†				
GMFCS level II		67·3 (28·0), n=51	78·8 (22·2), n=46	3·7 (2·0 to 5·2, p<0·0001)
GMFCS level III		54·6 (21·1), n=85	69·7 (23·3), n=81	7·3 (6·0 to 8·7, p<0·0001)
**CP-QoL Child domains**‡				
Social wellbeing and acceptance§				
	GMFCS level II	82·4 (15·3), n=51	84·7 (15·6), n=49	0·7 (−0·9 to 2·3, p=0·405)
	GMFCS level III	81·5 (12·7), n=82	82·1 (11·3), n=79	−0·01 (−1·1 to 1·1, p=0·979)
	All patients	81·8 (13·7), n=133	83·1 (13·1), n=128	0·3 (−0·7 to 1·2, p=0·580)
Feelings about functioning§				
	GMFCS level II	74·2 (16·4), n=51	81·2 (14·0), n=48	2·4 (0·6 to 4·2, p=0·008)
	GMFCS level III	68·1 (11·3), n=82	76·5 (12·1), n=79	3·4 (2·3 to 4·5, p<0·0001)
	All patients	70·5 (13·8), n=133	78·3 (13·0), n=128	3·0 (2·0 to 4·0, p<0·0001)
Participation and physical health§				
	GMFCS level II	59·1 (17·4), n=51	70·6 (18·8), n=49	4·1 (1·7 to 6·6, p=0·001)
	GMFCS level III	53·7 (16·6), n=82	63·6 (17·3), n=79	3·7 (2·0 to 5·5, p<0·0001)
	All patients	55·7 (17·1), n=133	66·3 (18·1), n=128	3·9 (2·5 to 5·3, p<0·0001)
Emotional wellbeing and self-esteem§				
	GMFCS level II	77·8 (18·5), n=51	82·7 (16·7), n=49	1·5 (−0·4 to 3·5, p=0·121)
	GMFCS level III	78·5 (10·7), n=82	82·1 (11·3), n=79	1·1 (−0·1 to 2·3, p=0·072)
	All patients	78·2 (14·2), n=133	82·3 (13·6), n=128	1·3 (0·2 to 2·3, p=0·018)
Access to services§				
	GMFCS level II	47·4 (13·0), n=51	49·7 (11·9), n=49	0·5 (−1·2 to 2·3, p=0·563)
	GMFCS level III	48·5 (11·3), n=82	52·1 (14·2), n=79	0·5 (−0·9 to 1·9, p=0·465)
	All patients	48·1 (11·9), n=133	51·2 (13·3), n=128	0·5 (−0·6 to 1·6, p=0·351)
Pain and impact of disability§				
	GMFCS level II	35·8 (17·8), n=51	23·9 (16·7), n=49	−1·7 (−3·4 to 0·1, p=0·06)
	GMFCS level III	36·8 (19·4), n=82	30·4 (16·8), n=78	−3·9 (−6·1 to −1·8, p<0·0001)
	All patients	36·4 (18·7), n=133	27·9 (17·0), n=127	−2·5 (−3·9 to −1·2, p<0·0001)
Family health¶				
	GMFCS level II	70·2 (19·6), n=51	79·9 (14·4), n=49	4·1 (2·2 to 5·9, p<0·0001)
	GMFCS level III	67·9 (18·4), n=81	69·9 (19·8), n=79	0·7 (−1·1 to 2·5, p=0·458)
	All patients	68·8 (18·8), n=132	73·8 (18·5), n=128	2·0 (0·7 to 3·4, p=0·003)

SDR=selective dorsal rhizotomy. GMFM-66=66-item Gross Motor Function Measure. GMFCS=Gross Motor Function Classification System. CP-QoL=Cerebral Palsy Quality of Life Questionnaire.

* There was evidence of an interaction between GMFCS level and GMFM-66 score (p=0·006).

† GMFM-66 centiles are GMFCS level-specific and, therefore, no aggregated score is calculated.

‡ Results are from the child primary caregiver or parent version.

§ No evidence of an interaction between GMFCS levels (p>0·05).

¶ There was evidence of an interaction between GMFCS level and CP-QoL Child family health domain (p=0·0127).

The CP-QoL results for all children improved significantly over 2 years for all domains assessed except social wellbeing and acceptance, emotional wellbeing and self-esteem, and access to services, although these did also improve slightly (table 2).

17 adverse events were reported for 15 children, with most having one event only (table 3). All but two were thought to be definitely or probably related to SDR, but none was a serious safety concern. The most common events were wound infection and persisting dysaesthesia in the feet and legs. No severe adverse events were reported and most resolved.

**Table 3:** Adverse events

	**Adverse event**			**Concomitant medication**	**Outcome**	**Additional information**
	Type	Intensity or severity	Related to SDR			
**Patient 1**						
Unknown	Uncovered dystonia	Mild	Unknown	Yes	Not resolved	Uncovered by SDR surgery
**Patient 2**						
400 days	Persisting dysaesthesia of feet and legs	Mild	Definitely	No	Resolved	Required hamstring lengthening 24 months after SDR
**Patient 3**						
30 days	Wound infection	Mild	Definitely	No	Resolved	··
**Patient 4**						
191 days	Persisting dysaesthesia of feet and legs	Mild	Definitely	No	Resolved	Treated with dabapentin
**Patient 5**						
2 days	Diarrhoea and vomiting	Mild	Unlikely	No	Resolved	Patient isolated and recovered quickly
**Patient 6**						
1 day	Constipation	Mild	Unlikely	No	Resolved with laxative	Related to pain medication
**Patient 7**						
22 days	Wound infection	Mild	Possible or likely	Yes	Resolved	Resolved with antibiotic treatment
**Patient 8**						
Unknown	Persisting dysaesthesia of feet and legs	Mild	Possible or likely	No	Not resolved	Hypersensitivity in right foot
**Patient 9**						
Unknown	Back pain	Mild	Possible or likely	Unknown	Resolved	··
**Patient 10**						
6 days	Wound infection	Mild	Definitely	No	Resolved	··
**Patient 11**						
55 days	Urgency	Mild	Unknown	No	Resolved	··
**Patient 12**						
28 days	Wound infection	Mild	Definitely	No	Resolved	··
**Patient 13**						
64 days	New weakness	Mild	Definitely	No	Resolved	··
**Patient 14**						
1 day	Urinary retention after removal of indwelling urinary catheter	Moderate	Definitely	No	Resolved	Also had previously implanted intrathecal baclofen pump removed at SDR surgery while tube remained in situ; catheter reinserted later
34 days	Persisting dysaesthesia of feet and legs	Mild	Definitely	No	Resolved	··
60 days	Swelling reported under wound site after discharge	Mild	Definitely	No	Resolved	Intrathecal baclofen pump removed at surgery
**Patient 15**						
Unknown	Granulation of wound	Mild	Definitely	Unknown	Resolved	··

Durations of adverse events are shown. SDR=selective dorsal rhizotomy.

The mean Gait Profile Score improved significantly from before to 24 months after SDR (p<0·001). Physiotherapists reported that the post-SDR physiotherapy recommendations had been implemented for 126 (95%) of 134 children at 24 months.

Five of the eight composite variables fulfilled the criterion for significance of p<0·0064, and even with adjustment for multiple testing, the primary outcomes were significant.

## Discussion

This prospective multicentre study followed up children with spastic diplegia for 24 months after they underwent SDR and found consistent improvements in patients' outcomes that increased annually over this period. In particular, GMFM-66 scores and normalised GMFM-66 centiles increased significantly with reasonably narrow 95% CIs. The estimated increase in GMFM-66 scores was higher in children classified as GMFCS level II than in those classified as GMFCS level III. All changes were greater than those that would be expected without SDR,^16^, ^17^, ^18^ validating the improvement in function. The findings were also consistent with a meta-analysis of RCTs, which showed a greater improvement in mean GMFM-66 with SDR plus physiotherapy than with physiotherapy alone with median follow-up of 12 months.^10^ Compared with the GMFM-66 centiles for children who had not undergone SDR, those in our study showed a similar trend towards an improvement from before to 24 months after SDR, with greater change seen in children classified as GMFCS level III than in those classified as GMFCS level II. Specifically, quality of life improved in terms of feelings about functioning, participation and physical health, emotional wellbeing and self-esteem, pain and impact of disability, and family health. The pain score was reduced by 2·5 units per year, which although a small change, was significant. These findings are consistent with previously reported improved quality of life 20–28 years after SDR^14^ and reduced pain after 17 years.^5^

No serious safety concerns related to SDR occurred in the 24-month postoperative follow-up period. SDR surgery and postoperative care have evolved since the RCTs,^6^, ^8^, ^9^ but they also showed no short-term safety concerns. Nevertheless, complications following SDR have been reported in other studies. Grunt and colleagues^7^ did a systematic review of 21 observational studies among which six studies reported spinal abnormalities, although no strong association was found with SDR. In a study with 10 years of follow-up, all 19 patients assessed had transient flexor spasm in the calves and hypotonia of the legs after SDR, one patient had transient urinary incontinence, and ten patients had hyperaesthesia.^27^

This study has several strengths. First, it represents a contemporary clinical series of prospectively enrolled patients with defined characteristics who underwent SDR in five paediatric neurosurgical centres. All the children underwent rigorous and standardised assessments before and after surgery. In 2017, NHS England requested an interim analysis of results to feed into policy discussions. All children were included in these analyses, which drew upon the primary outcomes and formed part of the evidence base used to support the introduction of SDR into specialised NHS commissioning in July, 2018, pending completion of the final analyses.^28^ Second, this study included a larger study sample than previous studies, which allowed prospective collection of a wide range of specific clinical data. Third, the primary outcomes of gross motor function and quality of life were measured with validated instruments that are widely used in research. Fourth, we used linear mixed models that account for the longitudinal measurements and allow the inclusion of all patients in the analyses despite a small number of missing values.

The main limitation of the study is the absence of a comparison group, which means that direct comparisons of outcomes cannot be made in children who did and did not undergo SDR. Given the evidence for the effectiveness of SDR from the 1990s,^6^, ^8^, ^9^ a further trial is unlikely to be acceptable and, therefore, no concurrent comparator patients can be recruited in England. To mitigate this limitation, we normalised our raw GMFM-66 scores based on an external standard. Another limitation is that the length of follow-up was 24 months, which does not allow assessment of outcomes into young adulthood. However, several long-term cohort studies with different groups of patients and outcomes suggest sustained (15–28 years) positive effects of SDR on quality of life^5^, ^14^ and function.^12^, ^13^

The clinical interpretation of the size of change in GMFM-66 is challenging because to our knowledge there is no established consensus on the minimum clinically important difference. The annual change of 2·3 GMFM-66 units we calculated is in broad agreement with the mean improvement in GMFM-66 reported by Bolster and colleagues,^29^ who found a change of 7·6 units after 5 years in 19 children. Further comparison is difficult due to differences in follow-up periods and populations of patients. However, this finding, along with the accompanying improvement in quality of life in our study (including reduction in pain) and improvement in gross motor function beyond that expected without SDR, strongly support the effectiveness of SDR.

Our study enrolled children aged 3–9 years with GMFCS levels II or III, which is estimated to occur in 15% of children with cerebral palsy and a diagnosis of spastic diplegia.^4^ The study was not designed to disentangle age effects or to extrapolate findings to children and young people of other ages or other GMFCS levels. There are also other unmeasurable factors, such as the quantity of physiotherapy received, which are confounders that cannot be accounted for. However, in this sense, our study reflects real-world clinical practice and provides a valuable picture of how the service would function and how patients would respond at least in the 24 months after SDR. NHS England has recommended that physicians continue to capture outcomes on this cohort of children.

In conclusion, this multicentre prospective study provided assessed current SDR practice with a standardised approach in 137 children and five centres. We could demonstrate consistent evidence of improvement in function beyond the expected trajectory of children with cerebral palsy not treated with SDR. This improvement in function was seen alongside clear improvement in quality of life, including pain reduction. The observed benefits were seen for children with GMFCS levels II and III cerebral palsy over a 2-year period and mirrored those reported in earlier RCTs. Finally, this study led directly to an interim national policy decision that SDR would be funded for eligible children with cerebral palsy in England from 2018.
