# Supplementary material for: Selective dorsal rhizotomy in ambulant children with cerebral palsy: an observational cohort study
Source: Lancet Child Adolesc Health. 2019 Jul;3(7):455–62. doi: 10.1016/S2352-4642(19)30119-1 (PMC7153769; doi:10.1016/S2352-4642(19)30119-1)
Supplement: Supplementary appendix [file mmc1.pdf]

THE LANCET
Child & Adolescent Health

Supplementary appendix

This appendix formed part of the original submission and has been peer reviewed. We post it as supplied by the authors.

Supplement to: Summers J, Coker B, Eddy S, et al, for the Selective Dorsal Rhizotomy Steering Committee. Selective dorsal rhizotomy in ambulant children with cerebral palsy: an observational cohort study. *Lancet Child Adolesc Health* 2019; published online April 29. http://dx.doi.org/10.1016/S2352-4642(19)30119-1.

Selective dorsal rhizotomy in ambulant children with cerebral palsy: an observational cohort study

Supplementary appendix

Table of contents

Page number

2	Membership of the Selective dorsal rhizotomy (SDR) Steering Committee and Participating NHS peadiatric neurosurgical centres in England, and funding
3-4	Commissioning criteria for post-operative physiotherapy
5-9	Literature search criteria, database search terms and PRISMA flowchart:<br>Table S1: Literature search criteria (PICO framework)
10	Figure S1: PRISMA flow diagram
11	Figure S2: Flow chart of recruitment for SDR
12	Table S2: Frequency distribution of percentage nerve rootlets cut
13	Figure S3: GMFM-66 individual observed trajectories for GMFCS level II
14	Figure S4: GMFM-66 individual observed trajectories for GMFCS level III
15	Figure S5: GMFM-66 centiles individual observed trajectories for GMFCS level II
16	Figure S6: GMFM-66 centiles individual observed trajectories for GMFCS level III
17	Table S3: Mean change in GMFM-66 per year associated with SDR and available normative and RCT data
18-19	Table S4: Secondary outcomes
20-35	Systematic review results/study overview:<br>Table S5: Summary of relevant studies and their specific methodologies<br>Figure S7: Cochrane risk of bias<br>Table S6: McLaughlin et al. (2002)[1] outcome summary<br>Table S7: McLaughlin et al. (2002)[1] main results
36	References

Membership of the Selective dorsal rhizotomy (SDR) Steering Committee

In addition to the listed authors, the full committee included:

Clinical/Physiotherapy representatives - Dr Ram Kumar, Paula Wilkins, Alison Burchell, Dr Guy Atherton, Beth Kershaw-Naylor, Emmanuel Turton, Dr Lucinda Carr, Deepti Chugh, Annabelle Townsend, Helen Navarra, Rajib Lodh, Alec Musson.

NHS England representatives - Anthony Prudhoe, Penelope Gray, Janette Harper, Robert Freeman.

National Institute for Health and Care Excellence representative – Lee Berry

Patient representatives – Sera Johnston, Sorcha Ford.

Participating NHS peadiatric neurosurgical centres in England

Alder Hey Children's NHS Foundation Trust

Great Ormond Street Hospital for Children NHS Foundation Trust

Leeds Teaching Hospitals NHS Trust

Nottingham University Hospitals NHS Trust

University Hospitals Bristol NHS Foundation Trust

Funding

This work was undertaken by King's Technology Evaluation Centre (KiTEC; Director Prof S Keevil) in partnership with National Institute for Health and Care Excellence (NICE) and National Health Service (NHS) England. The work of KiTEC was funded by NICE and supported by the National Institute for Health Research (NIHR) Biomedical Research Centre based at Guy's and St Thomas' NHS Foundation Trust and King's College London. Patient treatment costs were funded by NHS England. The views expressed are those of the author(s) and not necessarily those of the NHS, the NIHR or the UK Department of Health. JLP is a National Institute for Health Research (NIHR) Senior Investigator.

Commissioning criteria for post-operative physiotherapy

The following two boxes are extracted from the appendix of the National Health Service England Commissioning Board's Clinical Commissioning Policy Statement for Selective Dorsal Rhizotomy.[2]

Appendix 1.

Physiotherapy Post Selective Dorsal Rhizotomy
Acute Setting

Pre-Operative Assessment - Out patient service
1.0 hour OP combined clinic
4.0hrs physiotherapy evaluation including admin/liaison, ROM, MAS, Strength, Function, GMFM, GAIT
0.75 hrs MDT
2.5 hour pre operative assessment (second GMFM/community liaison/combined orthotics appointment)
Total 8.25 therapy hours – 0.22 WTE

Post Operative in patient stay
3.5 hrs per day for 15 days. Includes twice daily therapy session,
Any specialist assessments e.g. combined with orthotists/orthopaedic surgeon
Community liaison
Teaching to parents/carers required during the inpatient stay.
Some centres may offer this as an in-patient or outpatient service depending on the setting and stage of the child rehabilitation pathway.
Total - 17.5 hours per patient per week – 0.47 WTE

Post Operative Reviews
4 hours - therapy time at 4-6, 12 and 24 months – assessment, video, report and liaison
0.75 hours MDT discussion
Total - 0.14 hours per patient per week – 0.004 WTE

Teaching
6 hours per week – 0.16 WTE

TOTAL – WTE 0.85

Bandings range from 8a to 3 depending on the level of expertise required at each stage in the pathway.
Recommendations may vary according to each centres surgical technique.
Centre may be able to offer 'intensive' therapy blocks for 2 weeks three times a year to offer expert advise if community providers are unable to offer an increase of therapy post operatively. This will need costing accordingly.

Post-operative Physiotherapy for Selective Dorsal Rhizotomy in the Community Setting

An improvement in GMFM post SDR surgery is dependent on the access to post-operative physiotherapy. The recommendations are for guidance only and local provision may vary according to access to local services and a child's GMFCS level and will require further investigation with community physiotherapy teams.
The below provision will be in **addition** to a child's current local physiotherapy provision e.g. annual assessments for equipment, quarterly orthotic review, orthopaedic assessment, annual lower limb assessment and wheelchair assessment.

GMFCS Level II

- Hospital discharge to 4 months post-op: 2 times per week
- 4 to 6 months post-op: once per fortnight
- 6-12 months: once every 3-4 weeks
- 12-24 months post-op: monthly or as required

Therapy time for year one – 47.3 hours per child
Therapy time for year two - 12 hours per child
Total 0.03 WTE year 1
Total 0.006 WTE year 2

GMFCS Level III

- Hospital discharge to 4 months post-op: 3 times per week
- 4 to 6 months post-op: once per week
- 6-12 months: once per fortnight
- 12-24 months post-op: once per 2-4 weeks or as required

Therapy time for year one – 73.1 hours
Therapy time for year two - 25.8 hours
Total 0.04 WTE year 1
Total 0.01 WTE year 2

Current evidence for children having a pre op intensive therapy programme to improve recovery time post op. Children would benefit from a 6 week block of therapy preoperatively to improve muscle strength.
Therapy teams from UK centres offering SDR in England will review the child at 4-6 months, 12 months and 24 months. Local services may then adjust frequency of intervention based on these recommendations.
Community therapy providers may offer the same amount of therapy but deliver it in offer 'bock therapy session' depending on resources available.

Equipment Needs
Post SDR a child is likely to have a drop in function and therefore require access to additional equipment e.g. kaye walker/tripods/standing frame/orthotics.

Orthotics
Post SDR each child will require additional orthotic provision. As these children progress they will require a combined physiotherapy and orthotic review very 3-4 months

Literature Search Criteria, Database Search Terms & PRISMA flowchart

Table S1: Literature search criteria (PICO framework)

**Inclusion criteria**	
Population	Individuals with cerebral palsy<br>Subgroups of interest (based on inclusion criteria):<br>• Children (3 to 9 years)<br>• Spastic diplegic cerebral palsy<br>• GMFCS level II and III<br>• Dynamic spasticity in lower limbs affecting function and mobility<br>• MRI showing typical cerebral palsy changes and no damage to key areas of brain controlling posture and coordination[1]<br>• Mild to moderate lower limb weakness with ability to maintain antigravity postures
Intervention	Selective dorsal rhizotomy (SDR) (also known as functional posterior rhizotomy [FPR] or selective posterior rhizotomy [SPR])
Comparators	No treatment<br>Orthopaedic surgery<br>Antispasmodic muscle relaxant:<br>• Botulinum toxin (Botox)<br>• Tizanidine<br>Baclofen (intrathecal pump)<br>Phenol ('nerve deadeners')<br>Other comparators
Outcome	• GMFM-66<br>• GMFM-66 centiles<br>• CP-QoL Child (primary caregiver/parent)<br>• Adverse events<br>• Physiotherapy assessment<br>• Intraoperative assessment (i.e. nerve rootlets cut)<br>• Modified Ashworth Scale (MAS)<br>• Gait
Language restrictions	None
Search dates	If 1,000+ introduce search date restrictions of 1996+
**Exclusion criteria**	
Population	Subgroups of interest for exclusion when identifying comparable population groups:<br>• Presence of scoliosis<br>• Presence of hip dislocation (Reimer's index[3] should be <40%)<br>• Dystonia<br>• Genetic or neurological progressive illness<br>• Under 3 years of age, or older than 9 years<br>• GMFCS levels I, IV or V.<br>• Other medical or personal history of interest
Study design	Non-RCTs

[1]Typical MRI changes are those of white matter damage of immaturity, namely periventricular leukomalacia (PVL). Lesions in basal ganglia or cerebellum are contra-indications, since they are associated with other cerebral palsy types (dyskinetic/ataxia).

Database Search Terms

Cochrane Libraries

A Cochrane protocol for 'selective dorsal rhizotomy in the management of children with spastic cerebral palsy' is referred to in NICE's IP document [4]. However, the protocol referred to has been withdrawn from the Cochrane website (site accessed 11th April 2018). KiTEC sought clarification from Cochrane and the author(s). Cochrane replied: *'The protocol "Selective dorsal rhizotomy in the management of children with spastic cerebral palsy" was published in 2008 but the authors never move forward with the completed review. So, it was withdrawn by the Review Group in 2013, and then removed by the system.'* This was in accordance with Cochrane policy.

Search date 15th October 2018

ID	Search	Hits
#1	cerebral palsy	2,867
#2	cerebral pals*	2,899
#3	little*	29,145
#4	CP	9,673
#5	spastic*	1,893
#6	spastic diplegi*	199
#7	spastic quadriplegi*	71
#8	spastic hemiplegi*	220
#9	spastic monoplegi*	4
#10	rhizo*	481
#11	sensory nerve root* interrup*	15
#12	((function* or posterior or dorsal) adj rhizo*)	26
#13	sensory root* rhizo*	7
#14	sensory nerve root* rhizo*	5
#15	sdr	225
#16	1 or 2 or 3 or 4 or 5 or 6 or 7 or 8 or 9	42,133
#17	10 or 11 or 12 or 13 or 14 or 15	711
#18	16 and 17	135
#19	"trial":ti	226,857
#20	18 and 19	11

Embase

Search date 15th October 2018

Embase 1974 to 2018 Week 42

Ovid MEDLINE(R) Epub Ahead of Print, In-Process & Other Non-Indexed Citations, Ovid MEDLINE(R) Daily and Ovid MEDLINE(R) 1946 to Present

Global Health 1973 to 2018 Week 40

HMIC Health Management Information Consortium 1979 to July 2018

Maternity & Infant Care Database (MIDIRS) 1971 to August 2018

ID	Search	Hits
#1	cerebral palsy.mp	63,732
#2	cerebral pals*.mp	63,820
#3	little*.mp	1,287,703
#4	CP.mp	124,062
#5	spastic*.mp	70,369
#6	spastic diplegi*.mp	2,838
#7	spastic quadriplegi*.mp	1,371
#8	spastic hemiplegi*.mp	1,162
#9	spastic monoplegi*.mp	13
#10	rhizo*.mp	125,449
#11	sensory nerve root* interrup*.mp	0
#12	((function* or posterior or dorsal) adj rhizo*).mp	2,583
#13	sensory root* rhizo*.mp	3
#14	sensory nerve root* rhizo*.mp	0
#15	sdr.mp	4,037
#16	1 or 2 or 3 or 4 or 5 or 6 or 7 or 8 or 9	1,511,276
#17	10 or 11 or 12 or 13 or 14 or 15	129,091
#18	16 and 17	4,991
#19	trial.m_titl	476,548
#20	18 and 19	11

Pubmed

Search date 15th October 2018

ID	Search	Hits
#1	Search cerebral palsy	27,814
#2	Search cerebral pals*	25,295
#3	Search little*	572,330
#4	Search CP	68,472

#5	Search spastic*	26,401
#6	Search spastic diplegi*	1,164
#7	Search spastic quadriplegi*	541
#8	Search spastic hemiplegi*	492
#9	Search spastic monoplegi*	3
#10	Search rhizo*	42,293
#11	Search sensory nerve root* interrup*	0
#12	Search ((function* or posterior or dorsal)) AND rhizo*	6,283
#13	Search sensory root* rhizo*	1
#14	Search sensory nerve root* rhizo*	0
#15	Search sdr	2,709
#16	Search ((((((cerebral palsy) OR cerebral pals*) OR cp) OR spastic*) OR spastic diplegi*) OR spastic quadriplegi*) OR spastic hemiplegi*) OR spastic monoplegi*	111,845
#17	Search (((((rhizo*) OR sensory nerve root* interrup*) OR (((function* or posterior or dorsal)) AND rhizo*)) OR sensory root* rhizo*) OR sensory nerve root* rhizo*) OR sdr	9,054
#18	Search ((((((rhizo*) OR sensory nerve root* interrup*) OR (((function* or posterior or dorsal)) AND rhizo*)) OR sensory root* rhizo*) OR sensory nerve root* rhizo*) OR sdr)) AND (((((((cerebral palsy) OR cerebral pals*) OR cp) OR spastic*) OR spastic diplegi*) OR spastic quadriplegi*) OR spastic hemiplegi*) OR spastic monoplegi*	186
#19	Search trial[Title]	188,406
#20	Search (trial[Title]) AND ((((((((rhizo*) OR sensory nerve root* interrup*) OR (((function* or posterior or dorsal)) AND rhizo*)) OR sensory root* rhizo*) OR sensory nerve root* rhizo*) OR sdr)) AND ((((((((cerebral palsy) OR cerebral pals*) OR cp) OR spastic*) OR spastic diplegi*) OR spastic quadriplegi*) OR spastic hemiplegi*) OR spastic monoplegi*))	1

Web of Science

Search date 15th October 2018

ID	Search	Hits
#1	ts=(cerebral palsy)	46,297
#2	ts=(cerebral palsy*)	45,971
#3	ts=(little*)	1,113,605
#4	ts=(cp)	158,827
#5	ts=(spastic*)	43,633
#6	ts=(spastic diplegi*)	2,398
#7	ts=(spastic quadriplegi*)	1,313
#8	ts=(spastic hemiplegi*)	1,955
#9	ts=(spastic monoplegi*)	24
#10	ts=(rhizo*)	220,134
#11	ts=(sensory nerve root* interrupt*)	121
#12	ts=((function* or posterior or dorsal) NEAR rhizo*)	4,913

#13	ts=(sensory root* rhizo*)	619
#14	ts=(sensory nerve root* rhizo*)	451
#15	ts=(sdr)	7,578
#16	#9 OR #8 OR #7 OR #6 OR #5 OR #4 OR #3 OR #2 OR #1	1,342,643
#17	#15 OR #14 OR #13 OR #12 OR #11 OR #10	227,655
#18	#17 AND #16	8,471
#19	TI=(trial)	433,290
#20	#19 AND #18	27

Grey Literature

Search date 15th October 2018: the following sites: www.greylit.org/, www.opengrey.eu/, http://oaister.worldcat.org/, ntrl.ntis.gov/NTRL/. No ongoing trials for SDR were identified.

Figure S1: PRISMA Flow Diagram[5]

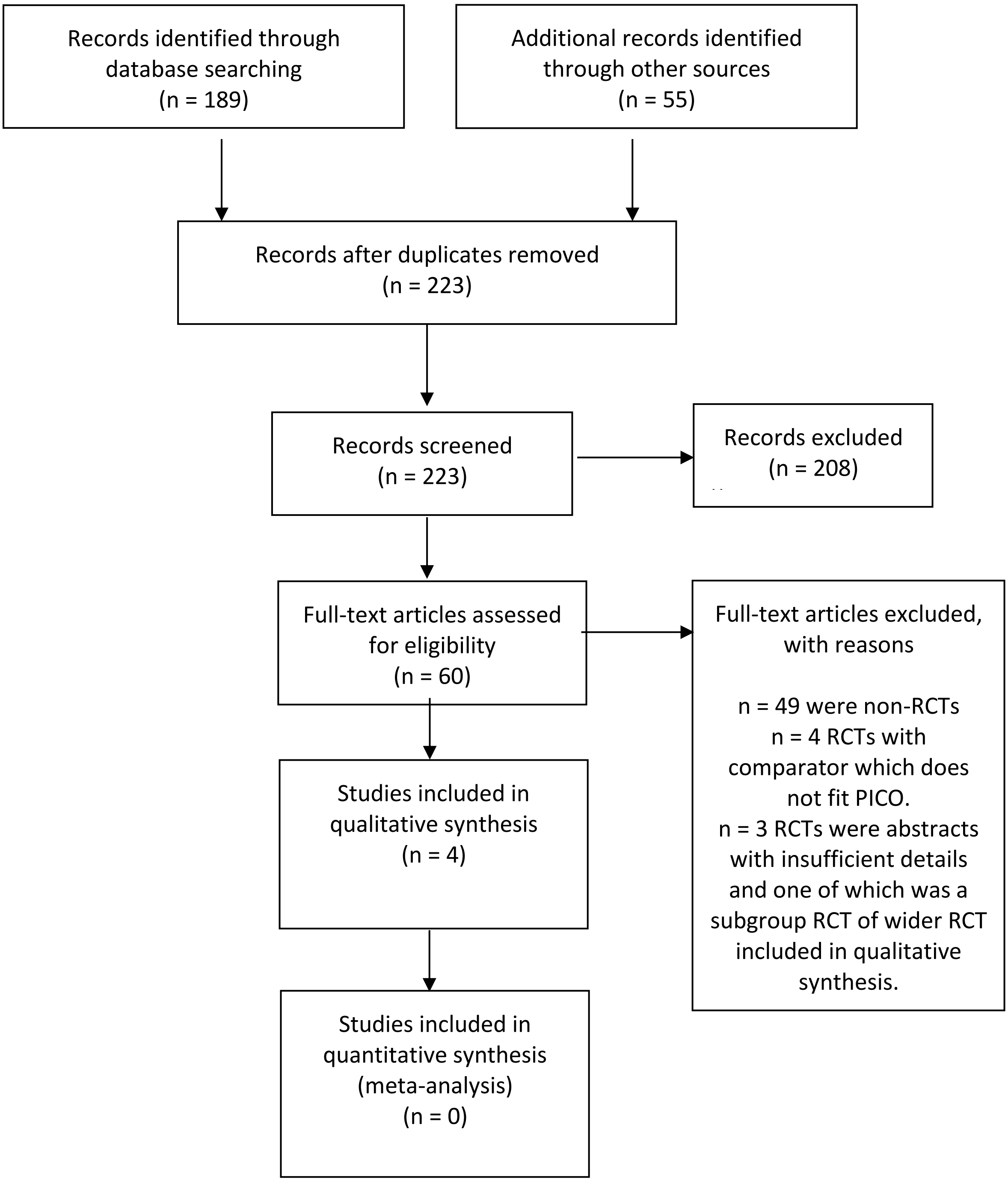

Figure S2: Flow chart of recruitment for SDR

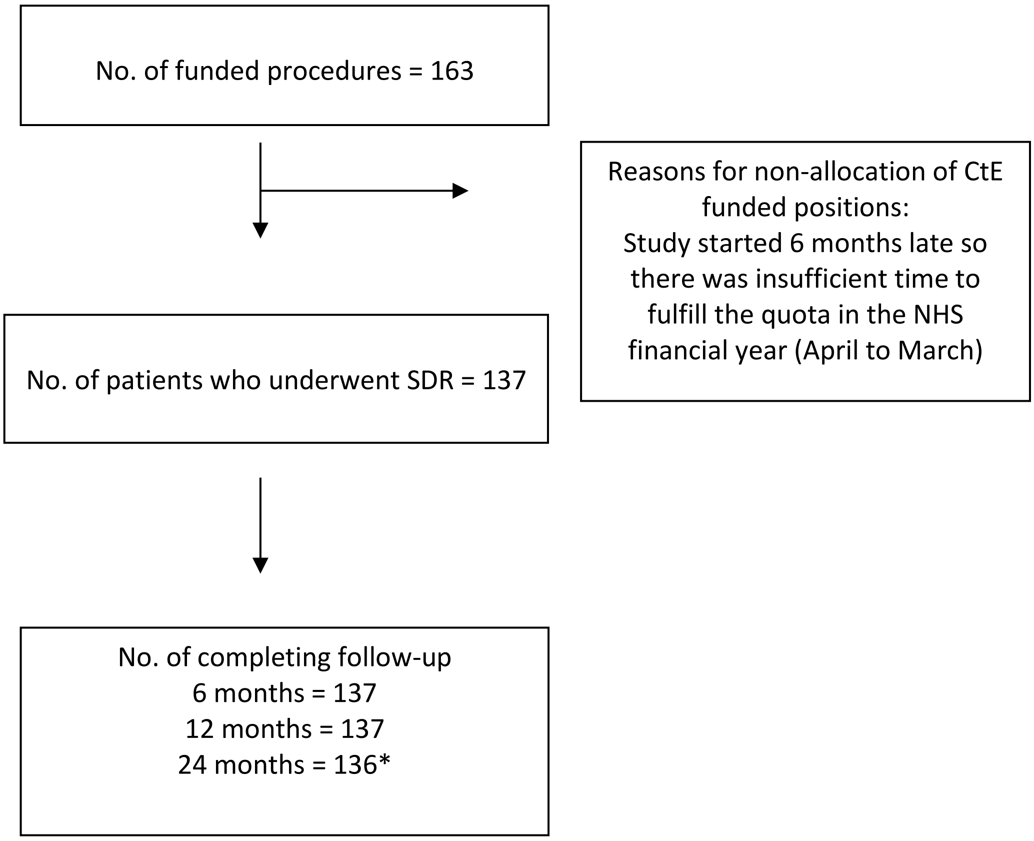

* One patient confirmed as lost-to-follow up at 24-month assessment.

Table S2: Frequency distribution of percentage nerve rootlets cut

Nerve rootlet	0%	1% to <50%	50% to <60%	60% to <70%	70% to <100%*	Total no. patients with >0% cut	Total no. patients
L1 left	19	0	30	76	0	106	125
L1 right	19	0	29	77	0	106	125
L2 left	0	2	8	124	3	137	137
L2 right	0	3	8	125	1	137	137
L3 left	0	1	14	121	1	137	137
L3 right	0	1	9	127	0	137	137
L4 left	0	0	10	126	1	137	137
L4 right	1	2	12	118	4	136	137
L5 left	0	2	13	81	41	137	137
L5 right	0	2	9	85	41	137	137
S1 left	3	5	7	77	45	134	137
S1 right	3	2	13	74	45	134	137

*No nerve rootlets were recorded with 100% cut.

Figure S3: GMFM-66 individual observed trajectories for GMFCS level II[6]

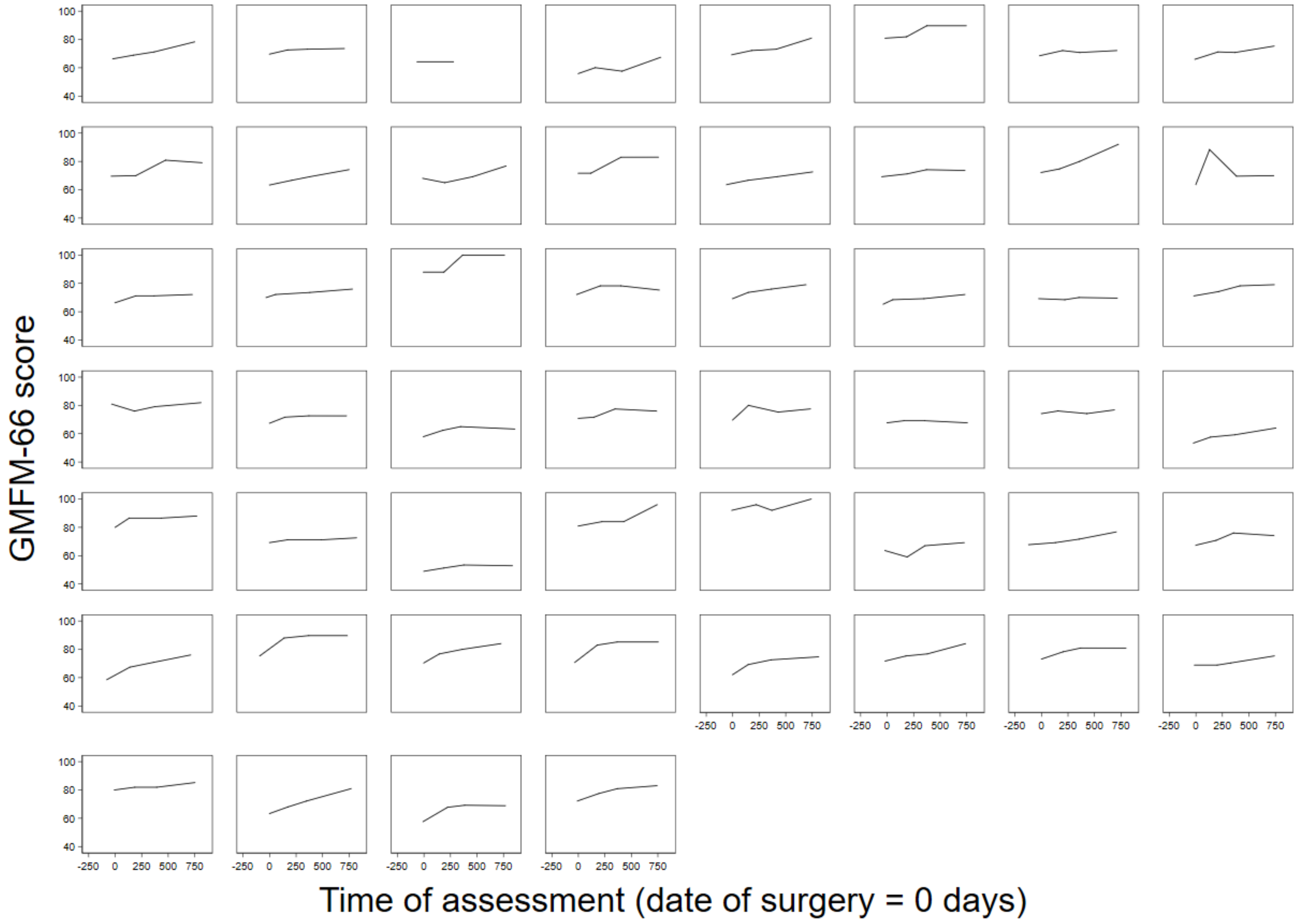

Figure S4: GMFM-66 individual observed trajectories for GMFCS level III

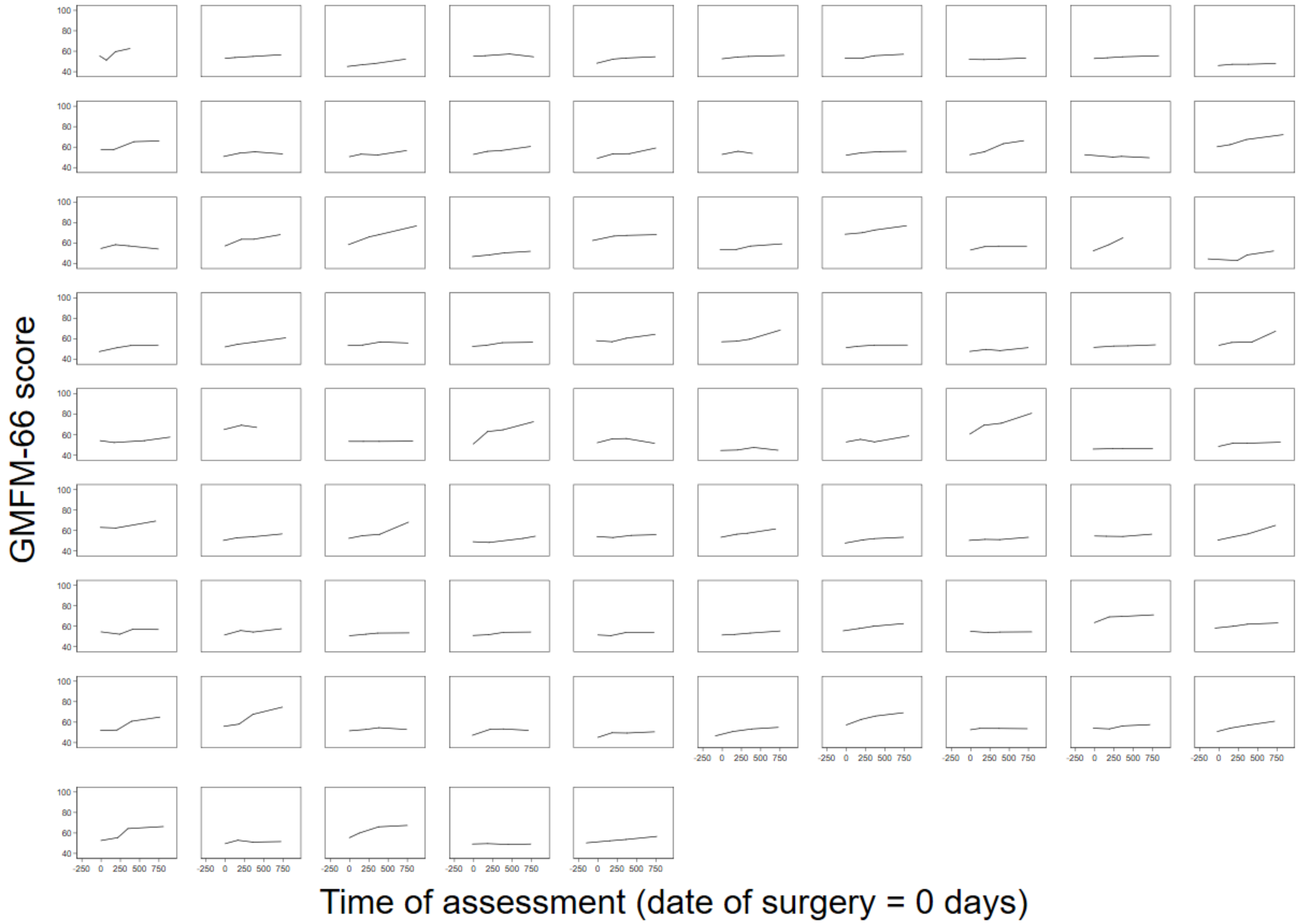

Figure S5: GMFM-66 centile individual observed trajectories for GMFCS level II

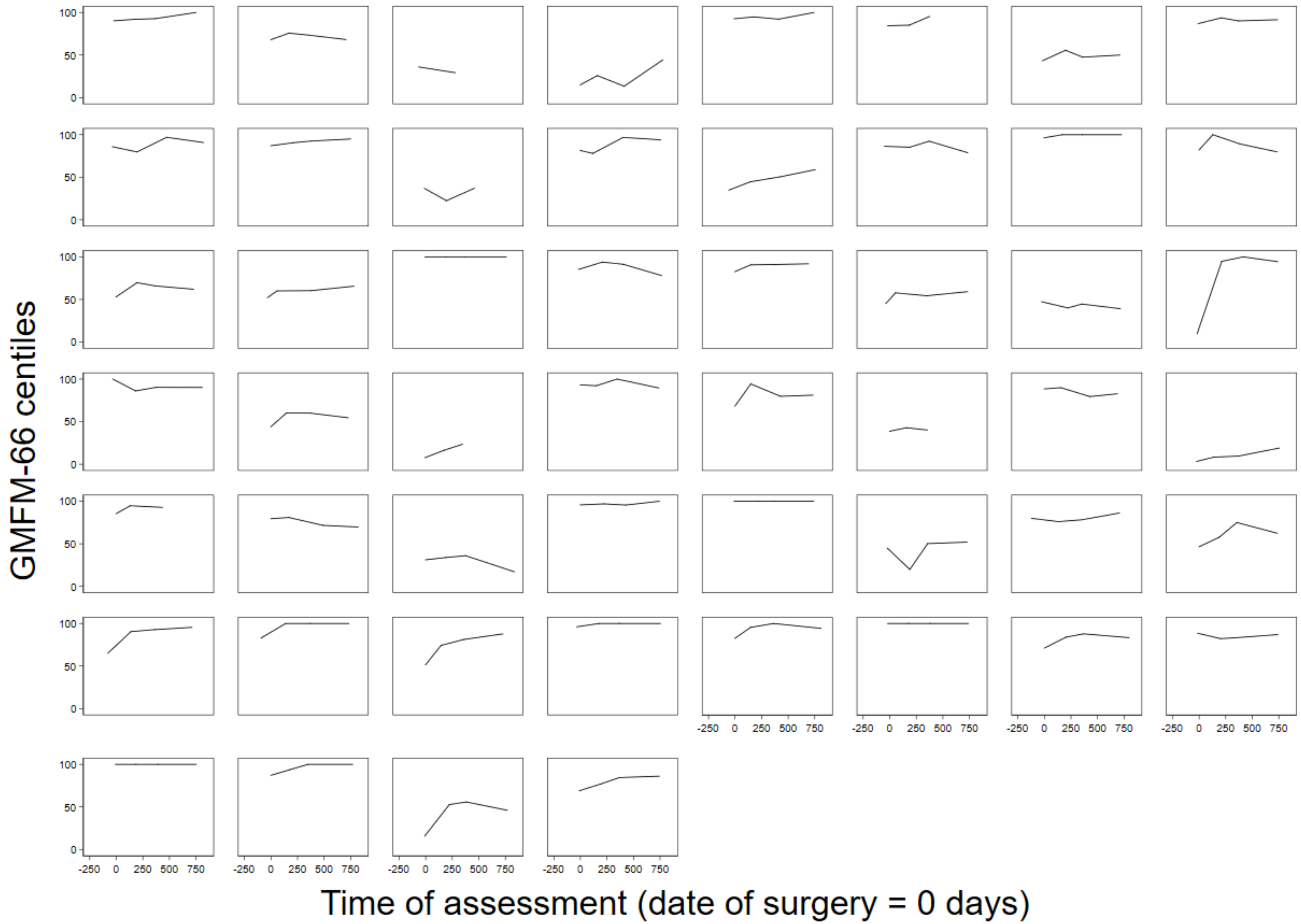

Figure S6: GMFM-66 centiles individual observed trajectories for GMFCS level III

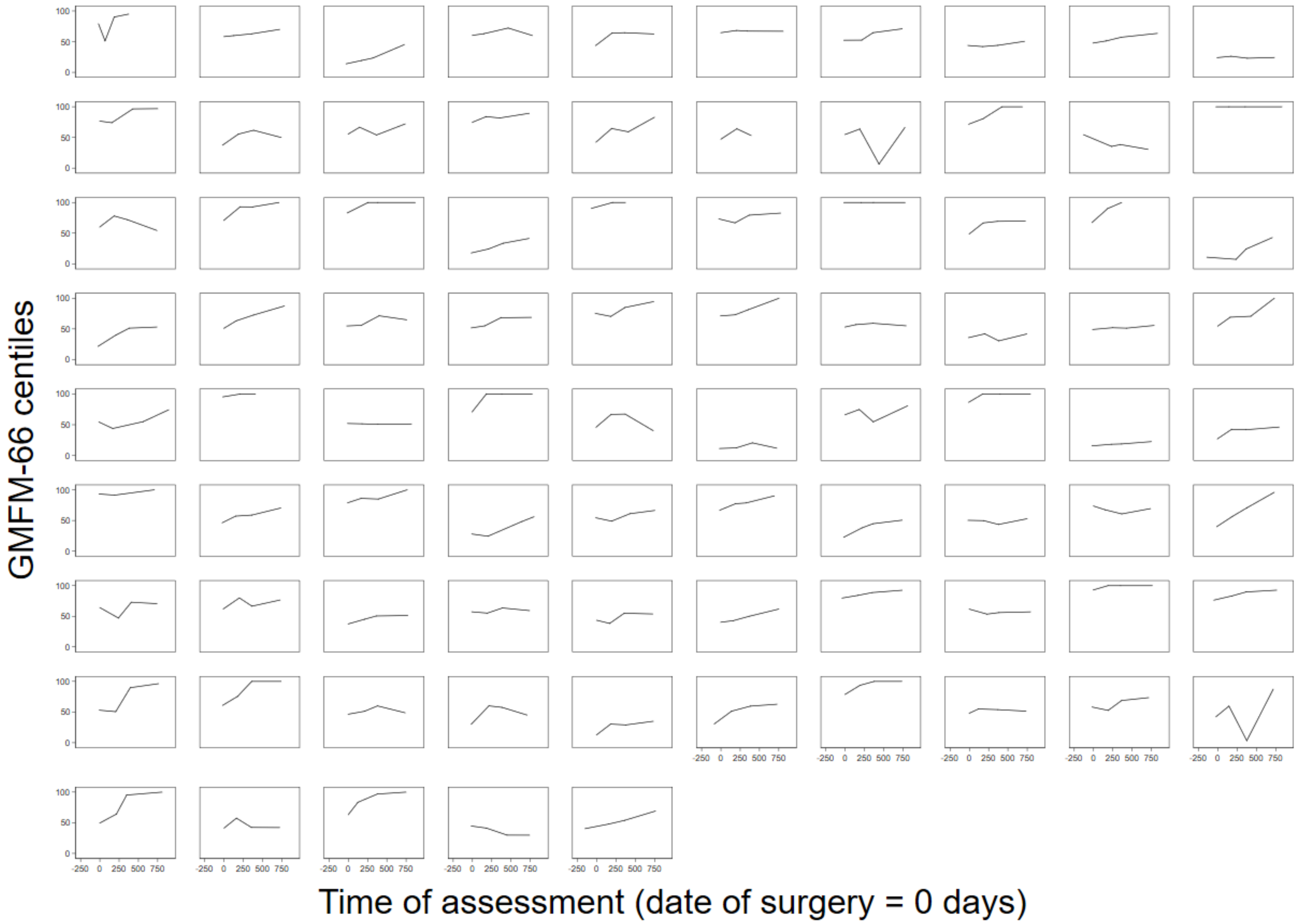

Table S3: Mean change in GMFM-66 per year associated with SDR and available normative and RCT data

Change in mean GMFM-66 per year	All children	GMFCS level II	GMFCS level III
SDR values from current study: Random effect mixed model estimates	3.23	3.78	2.88
Weighted CanChild norms[7]	1.9	2.2	1.7
Difference between SDR and control from the meta-analysis[1]	2.66		

Table S4: Secondary outcomes

Secondary outcomes (all children)	Pre-SDR	24 months post-SDR	Analysis
Modified Ashworth Scale (MAS)			
Adduction in neutral – no.			
Left	92	133	P<0.001
Right	92	133	P<0.001
Adduction in extension – no.			
Left	110	133	P<0.001
Right	113	133	P<0.001
Hamstring – no.			
Left	137	132	P<0.001
Right	137	132	P<0.001
Gastrocnemius – no.			
Left	137	133	P<0.001
Right	137	133	P<0.001
Gait			
Gait Profile Score – no., mean (SD)	108, 17.5 (5.6)	95, 13.5 (4.2)	P<0.001
Physiotherapy Assessment*			
Mobility Device – no./total no. (%)			
Posterior walker	89/251 (36)	70/255 (28)	N/A
Rifton pacer	3/251 (1.2)	1/255 (0.4)	N/A
Forward walker	5/251 (2.0)	9/255 (3.5)	N/A
Quad pods	8/251 (3.2)	9/255 (3.5)	N/A
Tripods	17/251 (6.8)	28/255 (11)	N/A
Crutches	4/251 (1.6)	11/255 (4.3)	N/A
Independent	33/251 (13)	38/255 (15)	N/A
Wheelchair	92/251 (37)	89/255 (35)	N/A
Orthotics Device – no./total no. (%)			

Secondary outcomes (all children)	Pre-SDR	24 months post-SDR	Analysis
Ankle foot orthosis (AFO)	105/314 (33)	85/263 (32)	N/A
Hinged AFO	12/314 (3.8)	9/263 (3.4)	N/A
Supramalleolar orthosis (SMO)	5/314 (1.6)	13/263 (4.9)	N/A
Boots	15/314 (4.8)	7/263 (2.7)	N/A
Insoles	3/314 (1.0)	15/263 (5.7)	N/A
Standard footwear	14/314 (4.5)	25/263 (9.5)	N/A
Gaiters	33/314 (11)	32/263 (12)	N/A
Specialist seating	68/314 (22)	40/263 (15)	N/A
Specialist standing	59/314 (19)	37/263 (14)	N/A
**Boyd and Graham (all children)**			
DorsiFlexion – Left – no.			P<0.001
0	16	3	
1	56	22	
2	40	29	
3	16	51	
4	8	24	
DorsiFlexion – Right – no.			P<0.001
0	15	2	
1	52	22	
2	36	32	
3	25	49	
4	8	24	

* Physiotherapists reported that many patients used multiple mobility and orthotic devices.

Systematic review results/study overview

We identified three RCTs[8-10] and one meta-analysis of the three RCTs[1] which fitted the criteria (see table S4).

Table S5: Summary of relevant studies and their specific methodologies

Reference & Study details	Overview/Methodologies	Key efficacy and safety findings	Comments
McLaughlin et al. (1998)[8]<br><br>Note: part of meta-analysis by McLaughlin et al. (2002)[1]<br><br>• RCT<br>• Seattle, USA<br>• n=43 patients.<br>• Patients ranged from 3 years to 18 years. This study therefore includes children outside the stated inclusion criteria however we were unable to extract information on those between 3 and 9 years of age at the time of SDR surgery.<br>• Hospitalisation ranged from 5 to 7 days and one surgeon performed all the surgeries.<br>• Patients randomised to either SDR plus physiotherapy (PT) or PT only group.<br>• Patients assessed at baseline, 6, 12 and 24 months.	• Of the 43 children who were enrolled there was no imbalance between the physiotherapy and physiotherapy and SDR group in terms of the following factors: gender, mean age at enrolment, age at start of treatment (not defined), ethnicity, gestational age, birthweight or cognitive ability. Six children withdrew from group assignment.<br>• Two of those six were originally in the physiotherapy group but requested to be part of the SDR group.<br>• One child in the PT group stopped participating after 6 months of physical therapy.	• Intention to treat and per protocol analyses were performed and they were '*statistically and clinically comparable*'.<br>• Only the per protocol analyses were presented.<br>• *'Several post hoc analyses were carried out on the GMFM data to search for sample subsets in which a difference favouring one of the treatment groups might be identified'.*<br>• The authors stated that *'children undergoing SDR made no more progress in functional mobility than children who received intensive PT without surgery'* and that '*there was sufficient statistical power to minimise the possibility we missed a statistically clinically important difference favouring SDR by chance alone'.*<br>• There was no evidence of a difference in the total GMFM-88 scores between the patients who had SDR and PT at 12 months (p=0.72) or at 24 months (p=0.94).<br>• Authors note that their *'results indicate that children undergoing SDR in our study made no more progress in functional mobility than the children who received intensive PT without surgery as measured by the GMFM'*.<br>• There was a difference of 1 grade (95% CI: -1.3 to -0.7) between the SDR and PT group at 12 months in comparison to baseline for the mean Ashworth scale in the major muscle groups in the lower extremities.<br>• At 24 months, the SDR+PT group exceeded the PT only group in mean reduction of spasticity by SMS measurement (-8.2 versus +5.1 newton meters/radian, p=0.02).<br>• The SDR+PT group and the PT only group demonstrated similar improvements in independent mobility on the GMFM score (7.0 versus 7.2 total percent score, p=0.94).<br>• The authors noted that *'the magnitude of change in the SDR and PT group in this study is no more than the average progress (6%) obtained*	• Authors conclude that '*Children undergoing SDR in our study made no more progress in functional mobility than children who received intensive PT without surgery, as measured by the GMFM*'.<br>• Unclear as to why some secondary outcomes measures were collected by investigators who were unmasked.<br>• Unclear how the Ashworth scale score was analysed, for example, the authors state that '*the mean Ashworth scale score for the major muscle groups in the lower extremities was reduced by one full grade in the SDR+PT group with no change*

<table>
<tr><th>Reference & Study details</th><th>Overview/Methodologies</th><th>Key efficacy and safety findings</th><th>Comments</th></tr>
<tr>
<td></td>
<td>

Used a ‘sample size large enough to detect a 10 percentage point difference in GMFM with at least 90% power using a two-tailed significance level of 0.05’.
At the time of publication, the clinical literature had no data regarding the placebo effect on the function of children undergoing SDR.
The authors noted that the ‘sham surgery was deemed unethical’ which prevented the use of a double masked design.
Investigators who had clinical contact with the children were not involved in the collection of primary outcome data and were masked to the results; ‘padded tape was placed over the lower back and covered with a shirt’

</td>
<td>
by children with cerebral palsy who received no specialist interventions over a 6-month period in the original validation sample’ (i.e. the original GMFM-88 paper).

The authors noted that ‘the intensity of the physical therapy may have masked the effect of SDR in the group comparison’.
There were no persisting sensory awareness or bladder control problems. Four children in the SDR & PT group suffered mild lower extremity paraesthesia for less than 8 weeks’ post-surgery. No long lasting sensory awareness or bladder control AEs were experienced.
The table below reproduces the reported GMFM change scores:

<table>
<tr><th colspan="5">Mobility outcomes: Gross Motor Function Measure change scores</th></tr>
<tr><th colspan="5">12 months</th></tr>
<tr><th></th><th>SDR+PT (n=21) Mean change (SD)</th><th>PT only (n=17) Mean change (SD)</th><th>Difference (95% CI)</th><th>P value</th></tr>
<tr><td>Lying/rolling</td><td>-0.01 (5.0)</td><td>0.83 (1.8)</td><td>-0.8 (-3.5 to 1.8)</td><td>p=0.53</td></tr>
<tr><td>Sitting</td><td>3.7 (13.2)</td><td>2.5 (7.9)</td><td>1.2 (-5.8 to 8.2)</td><td>p=0.73</td></tr>
<tr><td>Crawl/kneeling</td><td>2.8 (13.4)</td><td>2.9 (6.5)</td><td>-0.1 (-6.8 to 6.6)</td><td>p=0.98</td></tr>
<tr><td>Standing</td><td>10.1 (13.9)</td><td>7.5 (18.5)</td><td>2.6 (-8.4 to 14.0)</td><td>p=0.63</td></tr>
<tr><td>Walk/run/jump</td><td>7.8 (10.5)</td><td>7.3 (9.1)</td><td>0.5 (-6.0 to 7.0)</td><td>p=0.88</td></tr>
<tr><td>Total</td><td>4.9 (7.6)</td><td>4.2 (5.5)</td><td>0.8 (-3.5 to 5.0)</td><td>p=0.72</td></tr>
<tr><th colspan="5">24 months</th></tr>
</table>
</td>
<td>
in the PT only group (p<0.001) at 12 and 24 months’, however, from Table VI the median and range are presented and appear to have been analysed using a Wilcoxon Mann Whitney U test.

The authors report post-hoc subgroup analyses that were not stated a priori.

</td>
</tr>
</table>

Reference & Study details	Overview/Methodologies	Key efficacy and safety findings	Comments

before each child attended their data collection location.

- Children and families were reminded not to reveal their group allocation, however two breaks did occur and another member of staff performed data collection.
- The randomisation strategy employed was the sealed envelope technique.
- *'A 15 percentage point improvement on the GMFM total score'* was defined as a child who was very responsive to treatment.
- T tests were used for continuous variables, whilst a chi squared test or Fisher's exact test were used for categorical variables. Mann-Whitney U test was used *'where normal distributions could not be assumed'*.

	SDR+PT (n=21) Mean change (SD)	PT only (n=17) Mean change (SD)	Difference (95% CI)	P value
**Lying/rolling**	1.1 (2.9)	1.2 (3.7)	-0.1 (-2.2 to 2.1)	p=0.97
**Sitting**	4.6 (8.4)	6.2 (12.7)	-1.6 (-8.5 to 5.4)	p=0.65
**Crawl/kneeling**	4.4 (11.1)	4.7 (8.6)	-0.3 (-7.0 to 6.4)	p=0.93
**Standing**	9.9 (21.0)	13.3 (15.9)	-3.4 (16.0 to 9.1)	p=0.59
**Walk/run/jump**	12.4 (12.6)	10.8 (16.5)	1.6 (-8.0 to 11.0)	p=0.74
**Total**	7.0 (7.0)	7.2 (8.3)	-0.2 (-5.2 to 4.8)	p=0.94

- The table below shows the adverse events reported within the study. The authors noted that *'there were no serious adverse events'.*

**Summary of adverse events related to treatment**				
**Adverse event**	**SDR + PT**		**PT Only**	
	**Events**	**Children**	**Events**	**Children**
**Back pain**	14	6	0	0
**Lower extremity pain**	11	10	19	16
**Fatigue**	2	2	9	7
**Weakness**	5	4	5	3
**Urinary**	3	3	0	0
**Brace problem**	3	3	1	1
**Emotional/behavioral in PT**	7	6	13	6
**Other, musculoskeletal**	3	3	0	0
**Other, miscellaneous**	1	1	1	1

<table>
<tr><th>Reference & Study details</th><th>Overview/Methodologies</th><th>Key efficacy and safety findings</th><th>Comments</th></tr>
<tr><td></td><td>

- An adverse event (AE) questionnaire was completed every three months for 24 months. The severity, whether the AE was related to SDR and whether the AE was related to cerebral palsy were recorded for each AE, and importantly, each of these were defined a priori. To identify *'sensory changes a qualitative sensory examination of the lower extremities was performed at baseline and 24 months'*.

</td><td>

**Sensory**	4	4	0	0
**Total**	**53**	**20**	**48**	**17**

- The following table reports the results of the Ashworth scale analysis used to partially assess spasticity outcomes (along with Spasticity Management System [total path length and elastic path length {N m:rad}], not reported here):

**Spasticity outcome: Ashworth Scale change score**			
**12 months**			
**SDR + PT (n=21) Median (range)**	**PT only (n=17) Median (range)**	**Difference (95% CI)**	**P value**
-0.88 (-2.0 to 0)	0.13 (-1.0 to 1.0)	-1.0 (-1.3 to -0.7)	p<0.001
**24 months**			
**SDR + PT (n=20) Median (range)**	**PT only (n=17) Median (range)**	**Difference (95% CI)**	**P value**
-0.88 (-2.3 to -0.4)	0 (-1.0 to 1.3)	-1.0 (-1.4 to -0.7)	p<0.001

</td><td></td></tr>
<tr><td>

Steinbok et al. (1997)[9]

Note: part of meta-analysis by McLaughlin et al. (2002)[1]

- RCT, single-centre.
- Vancouver, British Colombia, Canada.
- n=30 children randomised to either SPR plus physiotherapy or

</td><td>

- Patients randomised to physiotherapy only group were later offered SPR. Randomisation was performed by *'independent party not involved with the care of the patient'*.
- Outcomes assessed included *'GMFM, Physiological Cost*

</td><td>

- The mean increase in total GMFM score from baseline to 9 months was reported as 11.3% (95%CI: 7.4 to 15.2) for the SPR group and 5.2% (95% CI: 3.1 to 7.2) for the control group, with a statistically significant difference of mean change of 6.1% (p=0.007).
- Authors noted all children in the control group went on to have SPR after the study finished.
- The following secondary outcomes were assessed using the change from baseline to 9 months in an independent t-test analysis:

**Assessment**	**SPR***	**Control***	**P value**
Physiological Cost Index	n=6 (m=-0.3, SD=0.15	n=5 (m=-0.27, SD=0.48)	p=0.89

</td><td>

- Method of calculating mean rootlet cut was not described.
- Raw GMFM scores for every child in both groups were reported. These are GMFM-88 scores.
- No paired t-test for within group GMFM total score from

</td></tr>
</table>

<table>
<tr><th>Reference & Study details</th><th>Overview/Methodologies</th><th>Key efficacy and safety findings</th><th>Comments</th></tr>
<tr>
<td>physiotherapy only. Two patients dropped out (one in each group).
• Children in the SPR group were aged 35 to 75 months (mean 50 months, median 47 months), and children in physiotherapy only group (control) were aged 35 to 77 months (mean 47 months, median 42 months).
• Assessed at baseline, 3, 6 and 9 months.
• For children who underwent SPR, mean posterior root cuts were 58% for L2, L3, L5 and S1. Mean rootlet cut for L4 was 42% and mean rootlet cut for S2 was 40%.
• For children who underwent SPR, discharge from hospital occurred on the 6th day post-SPR, and mobilization begun after 48 hours of bed rest.</td>
<td>Index, Peabody Fine Motor Scale, self-care assessment score and 10 measures of range, spasticity and strength'.
• Authors noted no significant difference between the two groups at baseline.
• Total no. of hours of physiotherapy for SPR groups averaged 81.8 hours (range 72 to 90 hours) and for control group averaged 81.3 hours (range 70 to 89 hours). Authors reported that the control group received physiotherapy within one month of being assigned, and received the same amount and type of physiotherapy as the SPR group.
• Children were dressed in one-piece leotards for all physiotherapy sessions/assessments, so that physiotherapist was not made aware of</td>
<td>
<table>
<tr><td>Peabody Score</td><td>n=14 (m=22.4, SD=20.2)</td><td>n=14 (m=17.4, SD=15.4)</td><td>p=0.48</td></tr>
<tr><td>Self-care assessment score</td><td>n=14 (m=10.5, SD=10.1)</td><td>n=14 (m=11.5, SD=7.5)</td><td>p=0.78</td></tr>
<tr><td colspan="4">Spasticity (Ashworth)</td></tr>
<tr><td>Hip adductors</td><td>n=14 (m=-1.4, SD=0.6)</td><td>n=14 (m=-0.3, SD=0.6)</td><td rowspan="3">p<0.001</td></tr>
<tr><td>Knee flexors</td><td>n=14 (m=-1.1, SD=0.5)</td><td>n=14 (m=-0.1, SD=0.7)</td></tr>
<tr><td>Ankle plantar flexors</td><td>n=14 (m=-1.5, SD=0.6)</td><td>n=14 (m=0, SD=0.8)</td></tr>
<tr><td colspan="4">Range of motion (degrees)</td></tr>
<tr><td>Hip adductors</td><td>n=14 (m=15.8, SD=10.6)</td><td>n=14 (m=-3.3, SD=8.6)</td><td rowspan="3">p<0.001</td></tr>
<tr><td>Knee flexors</td><td>n=14 (m=15.6, SD=15.6)</td><td>n=14 (m=-2.1, SD=10.9)</td></tr>
<tr><td>Ankle plantar flexors</td><td>n=7 (m=18, SD=5.9)</td><td>n=2 (m=17.5, SD=14.1)</td></tr>
<tr><td colspan="4">Muscle strength (kg force)</td></tr>
<tr><td>Knee extensors</td><td>n=5 (m=0.2, SD=1.5)</td><td>n=5 (m=0.7, SD=1.5)</td><td rowspan="4">p=0.64</td></tr>
<tr><td>Hip abductors</td><td>n=5 (m=0.5, SD=1.2)</td><td>n=5 (m=-0.2, SD=0.6)</td></tr>
<tr><td>Hip extensors</td><td>n=5 (m=0.9, SD=1.0)</td><td>n=5 (m=0.5, SD=1.2)</td></tr>
<tr><td>Ankle dorsiflexors</td><td>n=5 (m=1.3, SD=1.1)</td><td>n=5 (m=0.6, SD=1.4)</td></tr>
</table>
*n=number of subjects assessed, m=mean change, sd=standard deviation

• The authors noted that 'no patient on the study was given additional therapies outside the prescribed study protocol'.
• No complications were reported for the control (physiotherapy only) group.
</td>
<td>baseline to 9 months was provided.
• Ashworth scale score was analysed as a continuous variable.
• Secondary outcomes were not reported with 95% confidence intervals.
• Adverse events are reported for both groups.</td>
</tr>
</table>

Reference & Study details	Overview/Methodologies	Key efficacy and safety findings	Comments
	the treatment group that child was in.<br>• Analysis consisted of t-tests for independent mean GMFM total score change (baseline to 9 months) between the two groups.<br>• Secondary outcomes with continuous data were analysed with t tests for independent means.<br>• Bonferroni correction for multiple corrections was used when comparing one measure each of spasticity (hip adductors), range of motion - ROM (hip abduction) and muscle strength (knee extensors).	• One post-operative infection (spinal epidural abscess) and one case of transient urinary retention which lasted to the 4th day post-SPR were reported. There also one report of back pain in the SPR group (duration of 2 days and occurred 9 months after SPR).	
Wright et al. (1998)[10]<br><br>Note: part of meta-analysis by McLaughlin et al. (2002)[1]<br><br>• RCT<br>• MacMillan Centre, Toronto, Ontario, Canada	• All children had individualised therapy goals pre-randomisation. Control group therapy goals remained unchanged to limit bias.	• The authors noted *'no major negative effects were detected following the SDR procedure. There were no complaints of sensory changes or bladder dysfunction'*. The authors noted that 'one child suffered from a urinary tract infection post operatively, this was associated with the indwelling Foley catheter'.<br>• There were no significant differences in the age and gender of the children between the groups.	• No GMFCS levels reported.<br>• Limited information about baseline characteristics are provided, for example, age when receiving SDR.

<table>
<tr><th>Reference & Study details</th><th>Overview/Methodologies</th><th>Key efficacy and safety findings</th><th>Comments</th></tr>
<tr>
<td>

24 children (10 females, 14 males) with spastic diplegic cerebral palsy. Mean age of 58 months.
Patients randomised to SDR and physiotherapy only groups. There were 12 children per group.
Outcomes were measured at baseline, 6 and 12 months for both patient groups.
‘The minimum age was 41 months and the maximum age was 91 months’.

</td>
<td>

Therapy goals for intervention group changed after SDR, created by inpatient/occupational therapist group at the centre.
The control group received equivalent physiotherapy and occupational therapy. However, the rhizotomy group received a 6-week post-operative in-patient therapy programme.
L2 to S2 were isolated. Once it was established that these rootlets were functional ‘they were subdivided along natural planes into between 2 and 6 rootlets’ by the size of the root.
The authors noted that ‘on average, approximately 50% of each root was divided’.

</td>
<td>

The authors reported that there was a ‘correlation between GMFM total baseline scores and GMFM total 12 months change scores (r=-0.32)’.
The main GMFM (88) scores are reproduced in the below table:

<table>
<tr><th colspan="3">GMFM scores (percentage points) by category for each group at baseline, 6 months and 12 month assessments</th></tr>
<tr><th></th><th>Control (n=12)</th><th>Rhizotomy (n=12)</th></tr>
<tr><td colspan="3">Baseline</td></tr>
<tr><td>GMFM dimension</td><td>Mean (SD)</td><td>Mean (SD)</td></tr>
<tr><td>Lie/roll</td><td>91.2 (8.3)</td><td>92.8 (9.4)</td></tr>
<tr><td>Sit</td><td>83.7 (16.1)</td><td>74.3 (22.2)</td></tr>
<tr><td>Crawl/kneel</td><td>71.1 (19.4)</td><td>62.9 (26.9)</td></tr>
<tr><td>Stand</td><td>19.6 (17.2)</td><td>21.8 (15.9)</td></tr>
<tr><td>Walk/run/jump</td><td>13.2 (14.2)</td><td>10.6 (8.2)</td></tr>
<tr><td>Total</td><td>56.5 (12.2)</td><td>51.9 (13.4)</td></tr>
<tr><td colspan="3">6 months</td></tr>
<tr><td>Lie/roll</td><td>95.9 (2.8)</td><td>94.4 (6.7)</td></tr>
<tr><td>Sit</td><td>85.6 (17.9)</td><td>87.9 (15.1)</td></tr>
<tr><td>Crawl/kneel</td><td>76.3 (15.8)</td><td>68.4 (24.0)</td></tr>
<tr><td>Stand</td><td>23.7 (12.1)*</td><td>30.1 (23.4)*</td></tr>
<tr><td>Walk/run/jump</td><td>114.5 (15.4)</td><td>14.8 (7.8)</td></tr>
<tr><td>Total</td><td>58.5 (10.7)</td><td>58.7 (13.5)</td></tr>
<tr><td colspan="3">12 months</td></tr>
<tr><td>Lie/roll</td><td>96.2 (3.1)</td><td>98.7 (1.9)</td></tr>
<tr><td>Sit</td><td>87.9 (15.8)</td><td>87.7 (15.2)</td></tr>
<tr><td>Crawl/kneel</td><td>76.9 (10.4)</td><td>77.3 (19.2)</td></tr>
<tr><td>Stand</td><td>27.1 (19.6)</td><td>33.1 (23.5)</td></tr>
<tr><td>Walk/run/jump</td><td>15.7 (17.1)*</td><td>23.4 (19.5)*</td></tr>
<tr><td>Total</td><td>60.9 (12.5)*</td><td>64.0 (13.2)*</td></tr>
</table>
*p<0.05 between groups
</td>
<td>

Assessed MAS as a continuous variable.
While no AEs appear to have been reported after the 12-month assessment one participant underwent `serial casting for tightened ankle plantar flexors 3 years post rhizotomy’
Wright et al. stated that ‘the increase in GMFM total scores was 12.1 percentage points in the RG [SDR + physiotherapy group] group and 4.4 percentage points in the CG [physiotherapy only group] (P=0.02)’ for their trial. However, as the physiotherapy programmes are different based on whether the child has SDR or not, the physiotherapy only group could be confounding these results, as they

</td>
</tr>
</table>

Reference & Study details	Overview/Methodologies	Key efficacy and safety findings	Comments
	• Patients received intravenous morphine and a urinary catheter for approximately 3 to 4 days' post-surgery. Patients were turned from side to side every 4 hours during this time.<br>• Physiotherapy began on the 2nd or 3rd day after surgery.		'*received two therapy sessions per week (approximately 120 minutes in total)*' while for the SDR group during their 6-week post-operative stay '*each child received a 45-minute PT [physiotherapy] session daily and a 45 minute OT [occupational therapy] session twice weekly'*.<br>• The authors state that as per Russell et al's 1989[11] guidelines, a 6 percentage point improvement in the total score or within a dimension was considered clinically important. However, we have been unable to identify where the 6-percentage point improvement in GMFM-88 total or domain score is stated as clinically meaningful within Russell et al's study.

<table>
<tr><th>Reference & Study details</th><th>Overview/Methodologies</th><th>Key efficacy and safety findings</th><th>Comments</th></tr>
<tr>
<td>McLaughlin et al. (2002)[1]

Note: All three papers selected for this meta-analysis have been included in this review.

• Meta-analysis of three RCTs.
• The three RCTs consist of Steinbok et al. (1997) (Vancouver), McLaughlin et al. (1998) [8] (Seattle), and Wright et al. (1998)[10] (Toronto).
• All three studies from Northern America.
• n=90 from three RCTs.</td>
<td>• Children with spastic diplegia received either ‘selective’ dorsal rhizotomy (SDR) plus physiotherapy (PT) (SDR+PT) or PT without SDR (PT-only).
• Assessments made at baseline, 3, 6, 9, 12 and 24 months.
• Common outcome measures were used for spasticity (modified Ashworth scale) and function (Gross Motor Function Measure [GMFM]).
• Baseline and 9- to 12-month outcome data were pooled (n=90).
• Regression analysis of modified Ashworth, GMFM-66, GMFM-88 change score by % dorsal root tissue transected.</td>
<td>• Pooled GMFM data revealed greater functional improvement with SDR+PT (difference in change score +4.0, p=0.008).
• Multivariable analysis in the SDR+PT group revealed a direct relationship between percentage of dorsal root tissue transected and functional improvement.
• The authors stated that ‘the results suggest that the decision whether or not to perform SDR on a similar child partly rests on whether or not an anticipated mean GMFM change score increment of 4 percentage points above the amount of change with non-invasive care justifies the time, effort, and risk’.
• Below table gives SDR RCT trial outcome summary:
<table>
<tr><th colspan="4">SDR RCT trial: outcome summary</th></tr>
<tr><th></th><th>Vancouver[12]</th><th>Toronto[10]</th><th>Seattle[8]</th></tr>
<tr><td>Children (n)</td><td>28</td><td>24</td><td>38</td></tr>
<tr><td>Interval (months)</td><td>9</td><td>12</td><td>24</td></tr>
<tr><td>Mean difference in Ashworth change scores</td><td>-1.1 (p<0.001)</td><td>-1.0 (p=0.002)</td><td>-1.0 (p=0.001)</td></tr>
<tr><td>Mean difference in GMFM change scores</td><td>6.1% (p=0.007)</td><td>7.7% (p=0.02)</td><td>0.2% (p=0.94)</td></tr>
</table>
• The table below gives the main multivariable analysis results:
<table>
<tr><th colspan="4">SDR multivariate analysis: main results</th></tr>
<tr><th></th><th>Change Scores</th><th>Standard error</th><th>p-value</th></tr>
<tr><td>Ashworth</td><td>-1.23</td><td>0.11</td><td>p<0.001</td></tr>
</table></td>
<td>• Used individual patient data (IPD).
• Unclear if random or fixed effect modelling used.
• All three studies included were based in Northern America.
• Adverse events not listed, and only comment is in discussion.
• Included studies with different follow-up timepoints (two at 12 months and one at 9 months).
• Authors appear to have muddled the terms ‘multivariate’ and ‘multivariable’. Despite stating ‘multivariate’, we believe they mean ‘multivariable’.
• Gives comparator table for physiotherapy protocols for both intervention and control groups across studies.</td>
</tr>
</table>

<table>
<tr><th>Reference & Study details</th><th>Overview/Methodologies</th><th>Key efficacy and safety findings</th><th>Comments</th></tr>
<tr><td></td><td></td><td>
<table>
<tr><td>GMFM-88</td><td>4.53</td><td>1.44</td><td>p=0.002</td></tr>
<tr><td>GMFM-66</td><td>2.66</td><td>0.82</td><td>p=0.002</td></tr>
</table>

‘Based on the lack of interactional effects in the multivariate model, no subgroup defined by baseline characteristics was identified for which SDR is particularly effective. This was confirmed by looking at mean effects within and across sites in subgroups defined posthoc (analysis not presented). Retrospective GMFCS classification of baseline severity was not related to outcome’.
Authors concluded that ‘SDR+PT is efficacious in reducing spasticity in children with spastic diplegia and has a small positive effect on gross motor function’.
Authors state that ‘the three original studies did not report any worrisome problems with adverse events’.

</td><td>

Reports both GMFM-88 and GMFM-66 scores. Details of the calculation of the GMFM-66 scores are not described fully.
Assigned GMFCS levels to children retrospectively based on clinical notes.
No assessment of risk of bias.
The authors state that the modified Ashworth scale was used, however, the Wright et al. (1998)[10] reported the Ashworth scale score.
The Modified Ashworth Scale is incorrectly referred to as the Ashworth scale for Steinbok’s et al.[9] study.
McLaughlin et al. states the Ashworth scale is used as a primary outcome for all three studies, however in all the original papers the

</td></tr>
</table>

Reference & Study details	Overview/Methodologies	Key efficacy and safety findings	Comments
			Ashworth/MAS is used as a secondary outcome.<br>• The Ashworth/MAS scale is treated as continuous in Table VII as it is analysed using ANOVA, however in Figure 1 it is analysed using Wilcoxon's test, which is used for data which has some form of ordering as it can be ranked. Furthermore, if MAS was indeed used, the coding for the 1+ category should have been stated.<br>• It is unclear whether backwards elimination has been performed correctly, or whether forwards selection has instead been performed. The following quote suggest that the authors have performed forward selection, as opposed to backwards elimination: '*Once*

Reference & Study details	Overview/Methodologies	Key efficacy and safety findings	Comments
			significant main effects were identified, two-way interactions among the included variables were evaluated.' While stepwise methods are commonly used there are problems with using them such as preventing the investigator from really thinking about the problem for example, as Copas and Long (1991)[13] are quoted by Harrell[14]: '*The choice of the variables to be included depends on estimated regression coefficients rather than their true values, and so X_j is more likely to be included if its regression coefficient is over-estimated than if its regression coefficient is underestimated*'.

We identified one review/meta-analysis of randomized controlled clinical trials [1] and three randomized controlled trial,[8-10] each of which had contributed to the identified meta-analysis reported within the review. The review was published in 2002 and was conducted prior to publication of the PRISMA publication standard. It did not report its search strategy and did not include a PRISMA flow chart. It is thus unclear whether it strictly meets the definition of a systematic review. The review included an individual patient data (IPD) meta-analysis but since this was conducted prior to the publication of the PRISMA-IPD statement, there was no statement in relation to statistical assessment of heterogeneity and no statement in relation to the use of fixed or random effects. The review had not conducted any risk of bias assessment of the contributing studies although there were statements within the review indicating that some of these aspects had been considered.

We used the Cochrane risk of bias tool to assess the three RCT studies[8-10] and found that in general they were well reported and had included fairly robust methods of randomization and allocation concealment (Figure S7). None of the three studies were clinician-patient masked but given the nature of the intervention under consideration this is unsurprising but nevertheless does have the ability to bias findings. All three studies had attempted to address this by using strict methods to ensure that the outcome assessment was done without knowledge of treatment assignment although one paper reported that it was clear to assessors which children had received surgery.

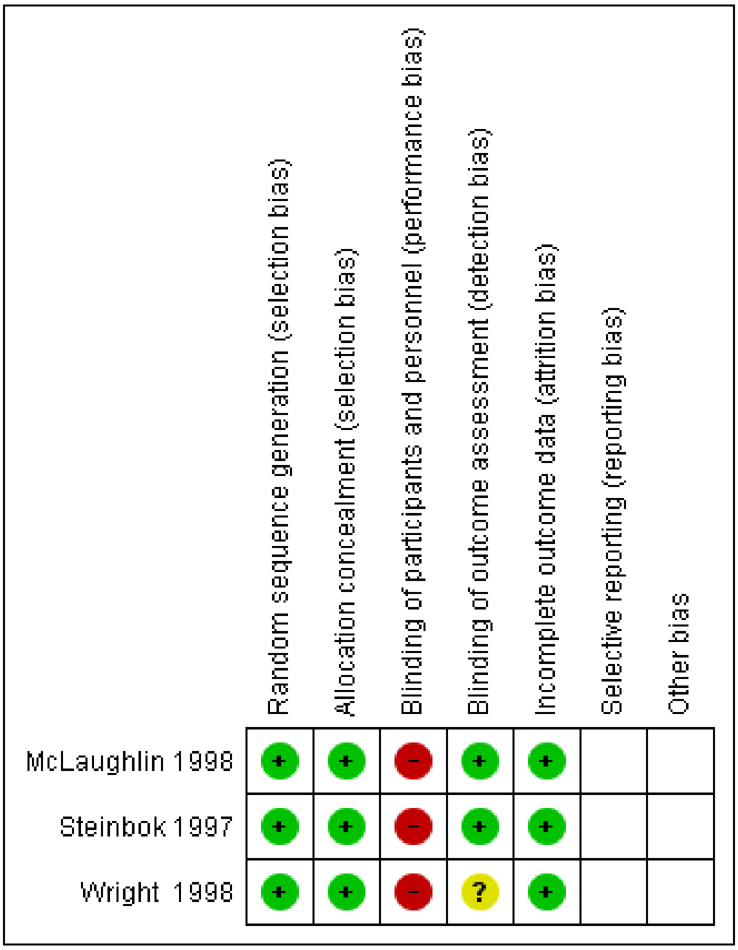

Figure S7: Cochrane risk of bias

We note that there are currently (as of October 2018) two systematic reviews registered with PROSPERO (International Prospective Register of Systematic Reviews, https://www.crd.york.ac.uk/prospero/) related to SDR. The first, due to be completed by the end of 2019, is investigating the long-term outcomes in children who undergo SDR (https://www.crd.york.ac.uk/prospero/display_record.php?RecordID=93544). The second is investigating both short and long-term outcomes following SDR in relation to gross motor function (https://www.crd.york.ac.uk/prospero/display_record.php?RecordID=91236).

We did not conduct a meta-analysis of the three RCT studies that were identified because none reported GMFM-66 and due to issues regarding the comparability of the study setting such as assessment timepoints, differing age cohorts and differences in baseline characteristics between the studies. For example, Steinbok et al. (1997)[9] and Wright et al.

(1998)[10] report a study population with lower GMFM scores at baseline in comparison to McLaughlin et al. 1998[8] and all three RCTs use different timepoints for assessments.

McLaughlin et al's (2002) review conducted additional analyses using raw data and used this to calculate the scores for GMFM-66 for the three RCTs listed above.[1] For this reason, we are reporting this review as the most up to date summary of available evidence and would highlight their findings. Included below for thoroughness, the original trial results for GMFM-88 and the GMFM-66 which is of relevance (Tables S6 and S7):

Table S6: McLaughlin et al. (2002)[1] outcome summary

**SDR RCT trial: outcome summary**			
	**Vancouver[12]**	**Toronto[10]**	**Seattle[8]**
Children (n)	28	24	38
Interval (months)	9	12	24
Mean difference in GMFM-88 change scores	6.1% (p=0.007)	7.7% (p=0.02)	0.2% (p=0.94)

Table S7: McLaughlin et al. (2002)[1] main results

**SDR multivariate analysis: main results***				
	**Change scores**	**Standard error**	**Anova F**	**p**
GMFM-88	4.53	1.44	9.92	0.002
GMFM-66	2.66	0.82	10.53	0.002

* 12 months' data used from Toronto and Seattle, and the 9-month data from Vancouver was used.

References

1. McLaughlin JF, Bjornson KF, Steinbok P, et al., Selective Dorsal Rhizotomy: meta-analysis of three randomized controlled trials. Dev Med Child Neurol, 2002. **44**: p. 17-25.
2. National Health Service England: Commissioning Board, Clinical Commissioning Policy Statement: Selective Dorsal Rhizotomy (SDR): Reference: NHSCB/E09/PS/a. 2013: United Kingdom.
3. Reimers J and Poulsen S, Adductor transfer versus tenotomy for stability of the hip in spastic cerebral palsy. J Pediatr Orthop, 1984. **4**(1): p. 52-54.
4. National Institute for Health and Care Excellence, Interventional Procedures Programme: Interventional procedure overview of selective dorsal rhizotomy for spasticity in cerebral palsy. 2006: Manchester, United Kingdom.
5. Moher D, Liberati A, Tetzlaff J, et al., Preferred Reporting Items for Systematic Reviews and Meta-Analyses: The PRISMA Statement. PLOS Medicine, 2009. **6**(7): p. e1000097.
6. Jann B, Customizing Stata graphs made even easier, in University of Bern Social Sciences Working Papers. 2018, University of Bern: Department of Social Sciences.
7. Russell DJ, Rosenbaum PL, Wright M, and Avery LM, Gross Motor Function measure (GMFM-66 & GMFM-88) User's Manual. 2nd Edition. Clinics in Developmental Medicine, ed. C. CanChild Centre for Childhood Disability Research. 2013, Canada: MacKeith Press.
8. McLaughlin JF, Bjornson KF, Astley SJ, et al., Selective Dorsal Rhizotomy: efficacy and safety in an investigator-masked randomized clinical trial. Dev Med Child Neurol, 1998. **40**: p. 220-232.
9. Steinbok P, Reiner AM, Beauchamp R, et al., A randomized clinical trial to compare posterior rhizotomy plus physiotherapy with physiotherapy alone in children with spastic diplegic cerebral palsy. Dev Med Child Neurol, 1997. **39**: p. 178-184.
10. Wright FV, Sheil EMH, Drake JM, et al., Evaluation of selective dorsal rhizotomy for the reduction of spasticity in cerebral palsy: a randomized controlled trial. Dev Med Child Neurol, 1998. **40**: p. 239-247.
11. Russell DJ, Rosenbaum PL, Cadman DT, et al., The gross motor function measure: a means to evaluate the effects of physical therapy. Dev Med Child Neurol, 1989. **31**(3): p. 341-352.
12. Steinbok P, Reiner A, and Kestle JRW, Therapeutic electrical stimulation following selective posterior rhizotomy in children with spastic diplegic cerebral palsy: a randomized clinical trial. Dev Med Child Neurol, 1997. **39**(515-520).
13. Copas, J.B. and T. Long, Estimating the Residual Variance in Orthogonal Regression with Variable Selection. Journal of the Royal Statistical Society. Series D (The Statistician), 1991. **40**(1): p. 51-59.
14. Harrell, F.E., Regression Modeling Strategies With Applications to Linear Models, Logistic Regression, and Survival Analysis. 2001, New York, USA: Springer.
